# Analysis of phenolic compounds in Parkinson’s disease: a bibliometric assessment of the 100 most cited papers

**DOI:** 10.3389/fnagi.2023.1149143

**Published:** 2023-05-02

**Authors:** José Messias Perdigão, Bruno José Brito Teixeira, Daiane Claydes Baia-da-Silva, Priscila Cunha Nascimento, Rafael Rodrigues Lima, Herve Rogez

**Affiliations:** ^1^Centre for Valorization of Amazonian Bioactive Compounds, Federal University of Pará, Belém, Brazil; ^2^Laboratory of Functional and Structural Biology, Institute of Biological Sciences, Federal University of Pará, Belém, Brazil

**Keywords:** bibliometric, Parkinson’s disease, phenolic compound, bibliometric and network analysis, neuroprotection

## Abstract

**Objective:**

The aim of this study was to identify and characterize the 100 most cited articles on Parkinson’s disease (PD) and phenolic compounds (PCs).

**Methods:**

Articles were selected in the Web of Science Core Collection up to June 2022 based on predetermined inclusion criteria, and the following bibliometric parameters were extracted: the number of citations, title, keywords, authors, year, study design, tested PC and therapeutic target. MapChart was used to create worldwide networks, and VOSviewer software was used to create bibliometric networks. Descriptive statistical analysis was used to identify the most researched PCs and therapeutic targets in PD.

**Results:**

The most cited article was also the oldest. The most recent article was published in 2020. Asia and China were the continent and the country with the most articles in the list (55 and 29%, respectively). *In vitro* studies were the most common experimental designs among the 100 most cited articles (46%). The most evaluated PC was epigallocatechin. Oxidative stress was the most studied therapeutic target.

**Conclusion:**

Despite the demonstrations in laboratorial studies, the results obtained point to the need for clinical studies to better elucidate this association.

## 1. Introduction

Parkinson’s disease (PD) is characterized by neurodegeneration and the presence of Lewy bodies, formed by α-synuclein (α-syn) fibrils, in the dopaminergic neurons (DNs) of the pars compacta of the substantia nigra of the brain (Balestrino and Schapira, 2020).

More than 10 million people worldwide are affected by PD. The prevalence of PD is approximately 0.3% in the general population, but this percentage increases to 1 and >4% in adults over 50 and 65 years, respectively (Balestrino and Schapira, 2020). The sum of the prevalences of PD in the 15 most popular nations in the world could reach 9 million people in 2030, approximately double the current prevalence, owing to population aging and advances in the treatment of PD (Dorsey et al., 2007).

The increase in prevalence influences the increase in the costs of the disease (Dorsey et al., 2007). Direct and indirect costs of PD are derived from drug and nondrug treatment, the payment of social security, the loss of productivity and income, hospitalizations, and laboratory tests. In the USA, the cost of the PD reached approximately USD 52 billion per year (Marras et al., 2018) while in Europe, the cost reached EUR 13.9 billion in 2010 (Gustavsson et al., 2011). In Japan, the direct cost per patient was approximately USD 37,994, and the indirect cost was approximately USD 25,356 (Yamabe et al., 2018).

Current drug treatment for symptom reduction or control includes dopaminergic pharmacological targets such as L-dopa; catechol-O-methyltransferase inhibitors; monoamine oxidase type B inhibitors (MAO-BIs); dopamine (DA) agonists; and non-dopaminergic pharmacological targets such as istradefylline, safinamide, clozapine, and amantadine (Poewe et al., 2017).

Unfortunately, the treatment of PD has side effects, such as impulsive and compulsive behaviors, nausea and hallucinations, due to the hyperstimulation of dopaminergic receptors and the serotonergic and cholinergic systems, which results in disturbances in the limbic and frontal cortical structures. In addition to side effects, drug treatment does not prevent disease progression (Connolly and Lang, 2014).

This fact has motivated the search for new substances and the development of neuroprotective drugs that prevent the death of DNs and delay the progression of the disease while causing fewer side effects (Reglodi et al., 2017). Furthermore, investing in treatments that delay disease progression by up to 20% could result in monetary benefits of USD 60,657 per patient (Johnson et al., 2013).

In this context, MAO-BIs (rasagiline and selegiline; Smith et al., 2015), coenzyme Q10 (Flint Beal et al., 2014), creatine monohydrate (Kieburtz et al., 2015), monoclonal antibodies directed against different parts of α-syn (Bergström et al., 2016), tocopherol, vitamin C (Etminan et al., 2005), docosahexaenoic acid (Cieslik et al., 2013) and phenolic compounds (PCs) have been gaining attention through demonstrations of their neuroprotective properties.

PCs are secondary plant metabolites that have at least one hydroxyl linked to an aromatic ring in their chemical structure, and they are synthesized by two metabolic pathways: the shikimate and/or acetate pathways (Cheynier et al., 2013). PCs can be classified according to their chemical structure mainly into flavonoids, phenolic acids, lignans, and stilbenes (Vuolo et al., 2018).

PCs can act on cellular mechanisms that cause DN degeneration through the modulation of gene expression and the activation of antioxidant enzymes regulated by the nuclear factor erythroid 2-related factor 2 (NrF2) pathway, thus suggesting great neuroprotective potential for PD (Magalingam et al., 2015; Hussain et al., 2018; Kujawska and Jodynis-Liebert, 2018).

The number of citations is a bibliometric parameter that indirectly indicates quality, impact, productivity and prestige (Baldiotti et al., 2021). Bibliometric analysis makes it possible to identify the most cited articles and, based on that, characterize the scientific production in the area of interest (Karobari et al., 2021). Bibliometric analyses on PD have already been performed (Li et al., 2008; Bala and Gupta, 2013; Robert et al., 2019; Pajo et al., 2020), but the role of PCs has not been addressed.

The identification and characterization of scientific production through bibliometric parameters could contribute to the understanding of the development and direction of research on PD and PCs. Thus, this study aimed to identify and characterize the 100 most cited papers on PCs in PD.

## 2. Materials and methods

### 2.1. Search strategy and database

The paper search was carried out using the Web of Science Core Collection (WoS-CC). The search terms are detailed in Table 1.

**Table 1:** Search strategy.

Database	Section
Web of Science	Core Collection
	Search strategy
	TS = (“Phenolic compound” OR “phenolic acid” OR “benzoic acid” OR “hydroxycinnamic acid” OR flavonoid OR anthocyanin OR flavanol OR flavonol OR flavanone OR flavone OR isoflavone OR tannin OR coumarin OR lignan OR quinone OR stilben OR curcuminoid OR provinol OR phenol OR polyphenol OR “polyphenolic antioxidant compound”) AND TS = (“Lewy Body” OR Parkinson OR “Parkinson Disease” OR Parkinsonism OR “neurodegenerative disease” OR synuclein)

Papers published up to June 2022 were searched with no restriction for language, publication year range, or methodology selection. Two researchers independently selected papers until the 100th most cited paper was found. Disagreements were resolved by the concordance method.

### 2.2. Data extraction

The articles were selected based on the following inclusion criteria: the words PD or PCs or their synonyms (Table 1) were present in the title and/or abstract and/or keywords, tests were carried out only with PD models, tests were carried out with natural PCs, and pure PCs or the major PCs (in the case of extracts) were identified and quantified. Conference papers and editorial papers were excluded.

The 100 most cited papers list was compiled in descending order based on the number of citations in the WoS-CC. In the event of a tie, the ranking was based on the highest citation density (the number of citations per year).

The article citation count, article title, publication year, study design, names of authors, the continent and country of origin, keywords, tested PC, tested therapeutic target and results of the top 100 most cited articles were recorded. The country of origin was determined by the published corresponding address.

### 2.3. Statistical analysis

Descriptive statistical analysis of the data extracted as described in the previous section was performed using the number of citations as the main variable. MapChart was used to represent the number of publications by country and continent. Articles were grouped according to the year of publication in 3-year periods.

Study designs were classified as follows: bibliographic studies, laboratory studies (*in vitro*, *in vivo*, *in situ*, and *ex vivo*) and observational studies. Furthermore, compounds were classified according to the subclass determined in the Phenol Explorer database (Rothwell et al., 2013).

### 2.4. Quantitative and qualitative analysis

Visualization of Similarities Viewer (VOSviewer) software was used to generate coauthor ship and author keyword co-occurrence cluster maps (van Eck and Waltman, 2010).

The analysis units used were author name in the coauthor ship cluster maps and author keyword in the co-occurrence networks. Author names were linked to each other based on the number of joint authors, and author keywords were linked by occurrence. Units were included when they appeared in at least one of the 100 most cited articles in both networks.

The terms were organized into clusters, with each cluster represented by a color. More important terms had larger circles, and strongly related terms were positioned close to each other. Moreover, lines were drawn between items to indicate relations, with thicker lines indicating a stronger link between 2 items (van Eck and Waltman, 2010).

## 3. Results

**Figure 1:**
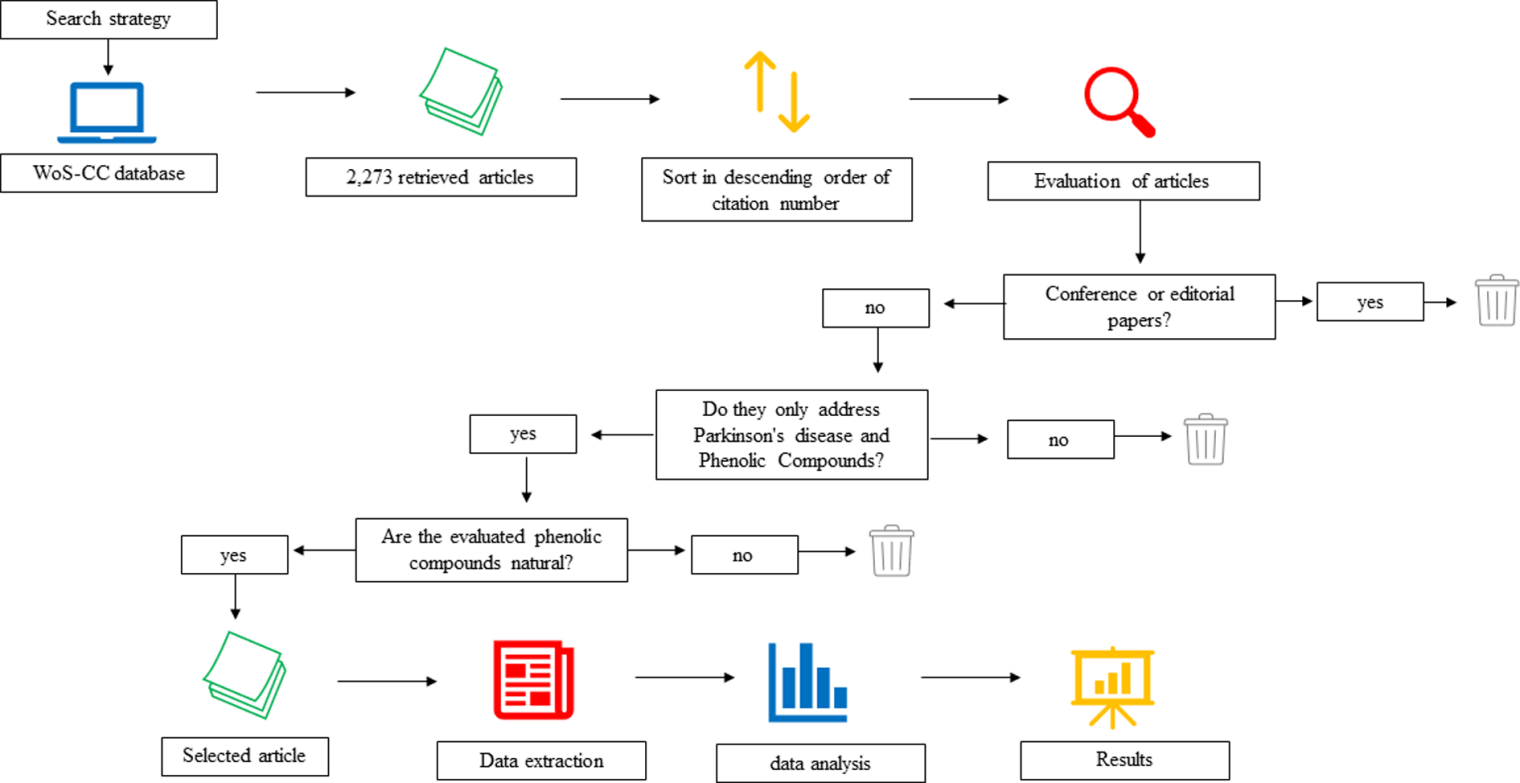
Methodological flowchart of the search, evaluation and selection of the most cited articles on PD and PC. The search for articles was carried out in WoS-CC, the retrieved articles were sorted in descending order based on the citation number in WoS-CC. The articles were evaluated with the eligibility criteria. Articles that did not address only PD and CP were excluded, as well as articles that did not address natural CP. 100 articles were selected and the necessary data for bibliometric analysis were extracted. Data were analyzed quantitatively and qualitatively using descriptive statistics. Software with VosViewer and Mapchart contributed to illustrate the results of the bibliometric analysis.

Through the search strategy used (Table 1), 2,273 articles were obtained. After listing the articles in descending order on the basis of the number of citations, 530 articles were screened by the eligibility criteria, of which 420 articles were excluded for not directly addressing PD and/or PCs (the titles of excluded articles can be found in the Supplementary material; Supplementary Table), resulting in the 110 most cited articles on PD and PCs. The 110 most cited articles were ranked from highest to lowest citation number. The first 100 articles were selected to compose the bibliometric analysis (Figure 1). The 100 most cited articles were ranked based on the number and density of citations in WoS-CC. The list of all articles and the data extracted for carrying out the bibliometric analysis are shown in Table 2.

**Table 2:** The 100 most cited articles on Parkinson’s disease and phenolic compounds.

R	Author (Reference)	Number of citations in the WoS-CC	Study design	Model	Pure compound(s) or extract (#)	Neuroprotective effects
1	Levites et al. (2001)	434	Laboratorial (*in vivo*)	Rats	EGCG	Decreased oxidative stress by increasing SOD and CAT activity, preventing DN loss and reducing TH levels
2	Zhu et al. (2004)	339	Laboratorial (*in vitro*)	Purified human α-syn	Baicalein	Inhibited the formation and disaggregation of α-syn fibrils
3	Levites et al. (2002a)	317	Laboratorial (*in vitro*)	SH-SY5Y cells	EGCG	Decreased oxidative stress through PKC stimulation and genetic modulation preventing cell death
4	Zbarsky et al. (2005)	306	Laboratorial (*in vivo*)	Rats	Curcumin Naringenin Quercetin Fisetin	Curcumin and naringenin decreased oxidative stress, preventing the reduction of TH positive cells and DA levels
5	Levites et al. (2002b)	245	Laboratorial (*in vitro*)	SH-SY5Y cells	#	Decreased oxidative stress through inhibition of nuclear translocation and NF-ĸB binding activity
6	Bureau et al. (2008)	203	Laboratorial (*in vitro*)	Neuronal PC12 cells and Microglial cell line N9	Resveratrol Quercetin	both prevented cell death by decreasing neuroinflammation
7	Caruana et al. (2011)	178	Laboratorial (*in vitro*)	Purified human α-syn	14 natural PCs and BTE	Baicalein, EGCG, myricetin, NDGA and BTE were classified as being the best combined inhibitors and disaggregators of α-syn fibrils
8	Mercer et al. (2005)	178	Laboratorial (*in vitro*)	Neuronal PC12 cells	Catechin Quercetin Chrysin Puerarin Naringenin Genistein	All attenuated the damage caused by oxidative stress in midbrain NDs. Catechin also reduced oxidative stress damage produced by hydrogen peroxide, 4-hydroxynonenal, rotenone and 6-OHDA
9	Mythri and Bharath (2012)	175	Bibliographic		Curcumin	Acted on oxidative/nitrosative stress, mitochondrial dysfunction and protein aggregation
10	Khan et al. (2010)	172	Laboratorial (*in vivo*)	Rats	Resveratrol	Increased antioxidant status; decreased DA loss; and attenuated the elevated level TBARS, PCa and PLA2 activity
11	Aquilano et al. (2008)	170	Bibliographic		#	Antioxidant and anti-inflammatory agents based on PCs were proposed for the treatment of PD
12	Meng et al. (2010)	164	Laboratorial (*in vitro*)	Purified human α-syn	48 flavonoids belonging to several classes	Eriodictyol, gossypetin, baicalein and 5,6,7,4′-tetrahydroxyflavone bound and stabilized α-syn in its native unfolded conformation
13	Gao et al. (2012)	160	Observational	Prospective cohort study	#	Men with high consumption of foods rich in flavonoids, mainly anthocyanins, were less likely to develop PD during 20–22 years of follow-up
14	Guo et al. (2005)	158	Laboratorial (*in vitro*)	SH-SY5Y cells	#	Modulated cell death by reducing oxidative stress from ROS sequestration, inhibition of PKC/ERK1/2 and NF-kB pathways, and gene modulation
15	Pandey et al. (2008)	156	Laboratorial (*in vitro*)	Recombinant wild-type α-syn	Curcumin	Inhibited α-syn fibril aggregation, disaggregated preforms and increased the solubility of α-syn fibrils in cells
16	Okawara et al. (2007)	151	Laboratorial (*in vitro*)	Organotypic midbrain slice cultures	Resveratrol	Prevented DNs death by decreasing oxidative stress and preventing GSH depletion and increasing propidium iodide uptake
17	Datla et al. (2001)	145	Laboratorial (*in vivo*)	Rats	Tangeretin	Showed evidence of crossing the BBB preventing TH cell loss and striatal DN content by reducing oxidative stress
18	Li et al. (2004)	144	Laboratorial (*in vitro*)	Microglia purified from rat; SH-SY5Y and Primary Mesencephalic Cultures	EGCG	Decreased neuroinflammation from inhibition of microglial secretion of NO and TNF-α through negative regulation of NO synthase and TNF-α expression
19	Wruck et al. (2007)	138	Laboratorial (*in vitro*)	PC12 and C6 cells	Luteolin	Decreased oxidative stress through activation of the Nrf-2 pathway
20	Hong et al. (2008)	132	Laboratorial (*in vitro*)	Purified human α-syn	Baicalein	Oligomers did not form fibrils even after a long time of incubation; they were globular species that were quite compact and extremely stable
21	Bournival et al. (2009)	131	Laboratorial (*in vitro*)	PC12 cells	Resveratrol Quercetin	Both decreased cell death through the reduction of oxidative stress that occurred from the modulation of the expression of the anti-apoptotic genes Bax and BCL2
22	Lou et al. (2014)	130	Laboratorial (*in vitro* and *in vivo*)	SH-SY5Y cells	Naringenin	Decreased oxidative stress from increased levels of Nrf-2 protein and activation of ARE pathway genes
23	Guo et al. (2007)	130	Laboratorial (*in vivo*)	Rats	#	Decreased oxidative stress, preventing increases in ROS and NO levels, lipid peroxidation, nitrite/nitrate content, inducible iNOS, and protein-bound 3-nitro-tyrosine
24	Chao et al. (2008)	125	Laboratorial (*in vitro*)	SH-SY5Y cells	Oxyresveratrol	Decreased oxidative stress demonstrated from reduced LDH release, caspase-3 activity, and ROS generation
25	Zhang Z. et al. (2012)	122	Laboratorial (*in vitro*)	PC12 cells	Baicalein	Increased transcriptional Nrf2/HMOX-1 expression and decreased Keap1, attenuating apoptosis, attenuating oxidative stress
26	Mu et al. (2009)	121	Laboratorial (*in vitro* and *in vivo*)	SH-SY5Y and PC12 cells and Rats	Baicalein	Attenuated muscle tremor by reducing oxidative stress, preventing cell death and neurite outgrowth
27	Karuppagounder et al. (2013)	120	Laboratorial (*in vivo*)	Rats	Quercetin	Prevented damage induced by oxidative stress, reduced unilateral rotations, reduced GSH activity, increased CAT and SOD activity, and regulated mitochondrial complex I activity
28	Lu et al. (2008)	118	Laboratorial (*in vivo*)	Rats	Resveratrol	Decreased oxidative stress by attenuating ROS generation, decreasing cell death and reducing motor deficit
29	Lorenzen et al. (2014)	117	Laboratorial (*in vitro*)	Purified human α-syn	EGCG	Inhibited α-syn fibril toxicity, moderately reduced membrane binding and immobilized the C-terminal tail of the oligomer
30	Zhang F. et al. (2010)	117	Laboratorial (*in vitro*)	Primary Rat Midbrain Neuron–Glia and Neuron-Astroglia, Microglia-Enriched Cultures	Resveratrol	Inhibited microglial activation and subsequently reduced pro-inflammatory factor release
31	Zhang et al. (2011)	113	Laboratorial (*in vitro* and *in vivo*)	PC12 cells and in zebrafish	Quercetin	Inhibited the overproduction of NO and the overexpression of iNOS and downregulated the overexpression of pro-inflammatory genes
32	Patil et al. (2014)	111	Laboratorial (*in vivo*)	Rats	Apigenin Luteolin	Both protected DNs, probably by reducing oxidative stress damage, neuroinflammation and microglial activation and enhancing neurotrophic potential
33	An et al. (2006)	111	Laboratorial (*in vitro*)	PC12 cells	Protocatechuic acid	Attenuated oxidative stress, increased cell viability and SOD and CAT activity, and decreased cell death
34	Nie et al. (2002b)	110	Laboratorial (*in vitro*)	PC12 cells	Catechin EGCG Epicatechin EGC Epicatechin gallate	EGCG or epicatechin gallate had greater effects on oxidative stress and led to inhibition of cell death, while other catechins had little effect
35	Jagatha et al. (2008)	108	Laboratorial (in sílico and *in vitro*)	N27 cells	Curcumin	Prevented cellular damage caused by oxidative stress, restored depleted GSH levels, protected against protein oxidation, and preserved mitochondrial complex I activity
36	Strathearn et al. (2014)	105	Laboratorial (*in vitro*)	Primary mesencephalic cultures	#	Extracts rich in anthocyanins and proanthocyanidins exhibited greater neuroprotective activity than extracts rich in other PCs
37	Kim et al. (2010)	102	Laboratorial (*in vitro* and *in vivo*)	SH-SY5Y and Primary mesencephalic cultures and Rats	#	Decreased oxidative stress from ROS regulation, NO generation, Bcl-2 and Bax proteins, mitochondrial membrane depolarization and caspase-3 activation and prevention of bradykinesia and ND damage
38	Nie et al. (2002a)	100	Laboratorial (*in vitro*)	PC12 cells	EGCG	Exerted significant protective effects against cell death induced by oxidative stress. EGCG was more effective than the GTP mixture
39	Zhang et al. (2015)	98	Laboratorial (*in vitro* and *in vivo*)	PC12 cells and Zebrafish	Protocatechuic acid Chrysin	When used in combination increased neuroprotective effects through a combination of cellular mechanisms of antioxidant and anti-inflammatory cytoprotection
40	Vauzour et al. (2010)	98	Laboratorial (*in vitro*)	Primary cortical neuron culture	Caffeic acid Tyrosol *p*-Coumaric acid	All induced neuroprotective effects related to the decrease in oxidative stress more powerful than those observed for flavonoids
41	Pan et al. (2003b)	94	Bibliographic		EGCG	Studies to understand biological activities and health benefits are still very limited. Further studies are needed to assess safety and efficacy in humans and determine neuroprotective mechanisms
42	Meng et al. (2009)	93	Laboratorial (*in vitro*)	Wild Type α-Syn and α-Syn Mutants purified	48 flavonoids belonging to several classes	Baicalein, eriodictoil, and 6-hesperidin were classified as strong inhibitors of α-syn fibrils
43	Tamilselvam et al. (2013)	90	Laboratorial (*in vitro*)	SK-N-SH cells	Hesperidin	Decreased oxidative stress from attenuation of loss of mitochondrial membrane potential; ROS generation; GSH depletion; improved CAT, SOD and GPx activities; upregulation of Bax, cyt C and caspases 3 and 9; and the downregulation of Bcl-2
44	Jimenez-Del-Rio et al. (2010)	90	Laboratorial (*in vivo*)	*Drosophila melanogaster*	Gallic acid Ferulic acid Caffeic acid Coumaric acid Propyl gallate Epicatechin EGC EGCG	The impairment of locomotor activity induced by oxidative stress was significantly recovered, although times of efficacy differed between compounds
45	Cheng et al. (2008)	90	Laboratorial (*in vivo*)	Rats	Baicalein	Inhibited oxidative stress causing increased DA, HVA and 5-HT levels and increased TH-ir neurons
46	Long et al. (2009)	89	Laboratorial (*in vivo*)	*Drosophila melanogaster*	Resveratrol	Improved oxidative stress-induced damage in climbing ability and lengthening average lifespan
47	Chen et al. (2008)	85	Laboratorial (*in vitro*)	Primary mesencephalic neuron–glia and microglia-enriched cultures	Luteolin	Attenuated the decrease in DA uptake and loss of TH-ir neurons from inhibition of neuroinflammation by decreasing excessive production of TNF-α, NO and SOD
48	Liu et al. (2008)	84	Laboratorial (*in vivo*)	Rats	Genistein	Prevented DN loss by reducing oxidative stress by increasing the expression of the BCL-2 gene
49	Molina-Jiménez et al. (2004)	79	Laboratorial (*in vitro*)	SH-SY5Y cells	Fraxetin Myricetin	Both restored the GSH redox ratio and decreased the oxidative stress
50	Magalingam et al. (2015)	75	Bibliographic		#	Gaps were identified in understanding the mechanism why flavonoids protect neuronal cells; few clinical studies showing evidence of the neuroprotection of PCs in patients with PD
51	Ye et al. (2012)	75	Laboratorial (*in vitro*)	PC12 cells	EGCG	Increased cell viability by decreasing oxidative stress through increasing mRNA expression of SOD1 and GPX1 and PGC-1α
52	Anusha et al. (2017)	73	Laboratorial (*in vivo*)	Rats	Apigenin	Decreased neuroinflammation through attenuated upregulation of NF-κB gene expression; inhibition of TNF-α, IL-6 and iNOS-1 release; prevented the reduction of mRNA expression of BDNF and GDNF
53	Cui et al. (2016)	72	Laboratorial (*in vivo*)	Rats	Curcumin	Alleviated motor dysfunction induced by oxidative stress, increased TH activity and GSH levels, reduced ROS and MDA content, and restored the expression levels of HMOX-1 and NQO1
54	Ardah et al. (2014)	72	Laboratorial (*in vitro*)	Purified human α-syn	Gallic acid	Binds to soluble oligomers with no β-sheet content and stabilized their structure
55	Bournival et al. (2012)	72	Laboratorial (*in vitro*)	Neuronal PC12 cells and Microglial cell line N9	Quercetin Sesamin	Both defended against neuroinflammation by preventing increases in IL-6, IL-1β and TNF-α mRNA and reduced expression of iNOS and mitochondrial superoxide radicals
56	Yu et al. (2010)	72	Laboratorial (*in vitro* and *in vivo*)	SH-SY5Y cells and Rats	Curcumin	Improved behavioral deficits induced by oxidative stress, enhanced the survival of TH^+^ neurons, inhibited the phosphorylation of JNK1/2 and C-Jun and cleaved caspase-3
57	Vauzour et al. (2008)	71	Laboratorial (*in vitro*)	Primary cortical neuron culture	Pelargonidin Quercetin Hesperetin Caffeic acid 4’-O-Me derivatives of Catechin Epicatechin	No effects on oxidative stress were observed with O-methylated flavan-3-ols. Concentrations above 0.3 μM of quercetin were toxic
58	Chaturvedi et al. (2006)	71	Laboratorial (*in vivo*)	Rats	#	Motor and neurochemical deficits induced by oxidative stress improved when BTE was given before 6-OHDA. Increased number of TH-ir neurons, TH protein level and TH mRNA expression in substantia nigra
59	Zhang Z. T. et al. (2010)	70	Laboratorial (*in vivo* and *in vitro*)	PC12 cells and Rats	Morin	attenuated behavioral deficits and ND death induced by oxidative stress
60	Sriraksa et al. (2012)	68	Laboratorial (*in vivo*)	Rats	Quercetin	Improved motor deficits induced by oxidative stress; increased DN density; increased SOD, CAT and GPx activity; and decreased AChE activity and MDA levels
61	Wang et al. (2005)	68	Laboratorial (*in vitro*)	Primary ventral mesencephalic neuron–glia cultures	Genistein	Attenuated neuroinflammation through inhibited microglial activation and the production of TNF-α, NO and superoxide
62	Kim et al. (2009)	67	Laboratorial (*in vitro*)	SH-SY5Y Cells	Naringin	Blocked JNK and P38 phosphorylation induced by inhibition of mitochondrial complex I, prevented changes in BCL2 and BAX expression, and reduced the activity of caspase 3 and the cleavage of caspase 9
63	Chen et al. (2015)	66	Laboratorial (*in vivo*)	Rats	Piceid	Attenuated motor deficits induced by oxidative stress; prevented the changes induced in the levels of GSH, thioredoxin, ATP, MDA and SOD in the striatum; and rescued DN degeneration
64	Liu et al. (2014)	66	Laboratorial (*in vitro*)	A53TαS purified	Gallic acid	Stabilized the extended native structure and interacted with α-syn transiently, inhibiting fibril formation
65	Zhang Z. J. et al. (2012)	66	Laboratorial (*in vivo* and *in vitro*)	PC12 cells and Zebrafish	Chrysin	Attenuated neuroinflammation and oxidative stress by decreasing IL-1β and TNF-α gene expression and inhibiting NO production and iNOS expression
66	Haleagrahara et al. (2011)	66	Laboratorial (*in vivo*)	Rats	Quercetin	Increase in DN levels through the attenuation of oxidative stress from the increase in GSH and decrease in the carbonyl protein content
67	Tapias et al. (2014)	64	Laboratorial (*in vivo*)	Rats	#	Increased oxidative stress leading to increased terminal nigrostriatal depression, loss of NDs and increased neuroinflammation from caspase activation
68	Lee et al. (2014)	64	Laboratorial (*in vivo*)	Rats	Baicalein	Reduction of neuroinflammation from the reduction of microglial activations, astrocytes, JNK and ERK; Improves motor skills and prevents the loss of DN
69	Kim et al. (2012)	64	Laboratorial (*in vitro*)	BV-2 and SH-SY5Y cells	Licochalcone E	Attenuated oxidative stress and neuroinflammation through modulation of the Nrf2-ARE system and upregulated downstream NQO1 and HMOX-1
70	Anandhan et al. (2012)	63	Laboratorial (*in vivo*)	Rats	Theaflavin	Regulated mitochondrial dysfunction by increasing TH and DA transporter expression and reducing caspase-3, 8, and 9
71	Chung et al. (2007)	62	Laboratorial (*in vitro*)	SH-SY5Y cells	EGCG	Potentiated cytotoxicity induced by oxidative stress generated by rotenone
72	Antunes et al. (2014)	60	Laboratorial (*in vivo*)	Rats	Hesperidin	Improved motor and behavioral deficits induced by oxidative stress; attenuated the reduction in GPx and CAT activity and TRAP and DA levels; and mitigated ROS levels and GSH activity
73	Ay et al. (2017)	59	Laboratorial (*in vivo* and *in vitro*)	MN9D cell and Rats	Quercetin	Induced the activation of PKD1 and Akt, increased mitochondrial biogenesis, improved behavioral deficits, and increased levels of TH-positive cells and DA
74	Kavitha et al. (2013)	58	Laboratorial (*in vivo*)	Rats	Mangiferin	Prevented behavioral deficits, oxidative stress, apoptosis, dopaminergic neuronal degeneration and DA depletion
75	Haleagrahara and Ponnusamy (2010)	58	Laboratorial (*in vivo*)	Rats	#	Reduced oxidative stress and the protein carbonyl content and increased the levels of SOD, CAT and GPx
76	Xu et al. (2017)	57	Laboratorial (*in vivo*)	Rats	EGCG	Rescued neurotoxicity by decreased oxidative stress; improved DA levels and substantia nigra ferroportin expression
77	Yabuki et al. (2014)	57	Laboratorial (*in vivo*)	Rats	Nobiletin	Rescued Motor and cognitive dysfunction by attenuation of oxidative stress
78	Roghani et al. (2010)	57	Laboratorial (*in vivo*)	Rats	Pelargonidin	Attenuated the rotational behavior, protected the neurons and decreased the oxidative stress
79	Hou et al. (2008)	57	Laboratorial (*in vitro*)	PC12 cells	EGCG	Attenuated apoptosis, maintaining mitochondrial membrane potential, inhibiting caspase-3 activity and downregulating the expression of SMAC
80	Ren et al. (2016)	56	Laboratorial (*in vivo*)	Rats	Dihydromyricetin	Attenuated behavioral impairments and ND loss, cell damage and ROS production induced by oxidative stress; increased phosphorylation of GSK-3 β
81	Zhu et al. (2013)	56	Laboratorial (*in vitro*)	Human wild-type α-syn	Quercetin Oxyquercetin	Oxidized quercetin species were stronger inhibitors of α-syn fibrils than quercetin
82	Molina-Jimenez et al. (2003)	56	Laboratorial (*in vitro*)	SH-SY5Y cells	Fraxetin Myricetin	Significantly decreased cytotoxicity generated by rotenone-induced oxidative stress, as well as LDH release, through the effect of fraxetine
83	Goes et al. (2018)	55	Laboratorial (*in vivo*)	Rats	Chrysin	Prevented behavioral changes; Modulated neuroinflammation through increased levels of TNF-α, IFN-γ, IL-1β, IL-2, IL-6 and NF-ĸB; and decreased levels of IL-10, DA, DOPAC, HVA and TH and TRAP
84	Cao et al. (2017)	54	Laboratorial (*in vivo* and *in vitro*)	SHSY5Y cells and Rats	Amentoflavone	Rescued DN loss; increased PI3K and Akt activation and the Bcl-2/Bax ratio; attenuated neuroinflammation through alleviated gliosis and levels of IL-1β and iNOS gene expression
85	Khan et al. (2013)	54	Laboratorial (*in vivo*)	Rats	Pycnogenol	Decreased stress oxidative; restored GSH levels and the activities of GPx, GSH and SOD; inhibited the expression of NF-ĸB and attenuated neuroinflammation through the release of COX-2, iNOS, TNF-α and IL-1β
86	Brunetti et al. (2020)	53	Laboratorial (*in vivo*)	*Caenorhabditis elegans*	Hydroxytirosol Oleuropein aglycone	Increased the survival after heat stress oxidative, but only hydroxytirosol could prolong the lifespan in unstressed conditions
87	Wu et al. (2015)	53	Laboratorial (*in vitro*)	BV-2 cells	Biochanin A	Decreased neuroinflammation through attenuated the mRNA expression of TNF-α and IL-1β; inhibited iNOS mRNA and protein expression and the phosphorylation of JNK, ERK and P38
88	Pan et al. (2003a)	53	Laboratorial (*in vivo* and *in vitro*)	Primary culture of embryonic mesencephalic and primary mensencephalic cells	#	Significantly attenuated the loss of TH-positive cells induced by mitochondrial dysfunction
89	Leem et al. (2014)	52	Laboratorial (*in vivo*)	Rats	Naringin	Increased GDNF levels in DA neurons, activated the mammalian target of rapamycin complex 1, and attenuated neuroinflammation by decreasing the level of TNF-α in microglia
90	Du et al. (2012)	52	Laboratorial (*in vivo*)	Rats	Curcumin	Prevented the decrease in the levels of DA and DOPAC and inhibited the decrease in TH ^+^ neurons and the numbers of iron ^+^ cells induced by oxidative stress
91	Xu et al. (2016)	51	Laboratorial (*in vitro*)	Synthetic α-syn	EGCG	Inhibited α-syn fibril aggregation through unstable hydrophobic bonds
92	Subramaniam and Ellis (2013)	51	Laboratorial (*in vivo*)	Rats	Umbelliferone Esculetin	Both significantly attenuated oxidative stress y in the substantia nigra pars compacta but not the striatum, prevented the increase in nitrosative stress and prevented caspase 3 activation but inhibited MAO activity
93	Kang et al. (2010)	51	Laboratorial (*in vivo* and *in vitro*)	HT22 hippocampal neuronal cells and Rats	EGCG	Inhibited the O-methylation of L-dopa and moderately reduced the accumulation of 3-O-methyldopa in plasma and striatum decreasing oxidative stress
94	Guan et al. (2006)	51	Laboratorial (*in vitro*)	PC12 cells	Protocatechuic acid	Prevented the formation of ROS, GSH depletion and the activation of caspase-3 and upregulatedBcl-2 induced by mitochondrial dysfunction
95	Jiang et al. (2010)	50	Laboratorial (*in vitro*)	E46K α-syn	Baicalein	Attenuated mitochondrial depolarization and proteasome inhibition and protected cells from toxicity as well as reduced α-syn fibrillation
96	Kujawska and Jodynis-Liebert (2018)	49	Bibliographic		#	Analyzed studies encouraged the search for phytochemicals exerting neuroprotective effects on DA neurons, and delaying their degeneration was found to be highly desirable
97	Macedo et al. (2015)	49	Laboratorial (*in vitro*)	Synthetic α-syn	Myricetin	Inhibited α-syn toxicity and aggregation in cells
98	Li et al. (2012)	49	Laboratorial (*in vitro*)	PC12 cells and rat brain mitochondria	Baicalein	Attenuated oxidative stress, suppressed apoptosis, inhibited the accumulation of ROS, alleviated ATP deficiency, and acted in mitochondrial membrane potential dissipation and caspase-3/7 activation
99	Park et al. (2008)	49	Laboratorial (*in vitro*)	SN4741 cells	Carnosic acid	Inhibited oxidative stress by preventing caspase-3 activation, JNK phosphorylation, and caspase-12 activation
100	Datla et al. (2007)	49	Laboratorial (*in vivo*)	Rats	Tangeritin Nobiletin Catechin Epicatechin Epicatechin gallate Formononetin Genistein	Pretreatment with plant extracts rich in catechins had no effect on oxidative stress of nigrostriatal NDs

R = ranking.

### 3.1. Oldest, newest and most cited article

The article “Green tea polyphenol (−)-epigallocatechin-3-gallate prevents N-methyl-4-phenyl-1,2,3,6-tetrahydropyridine-induced dopaminergic neurodegeneration. Journal of Neurochemistry. 2001 Sep; 78 (Yamabe et al., 2018): 1073–1082” is the most cited (434 citations) and the oldest on the top 100 list in the WoS-CC.

Additionally, on the same criteria, the most recent article on the list is “Healthspan Maintenance and Prevention of Parkinson’s-like Phenotypes with Hydroxytyrosol and Oleuropein Aglycone in *C. elegans*. International Journal of Molecular Sciences, 2020, 21,” with 53 citations.

### 3.2. Authors who contributed to the 100 most cited articles

**Figure 2:**
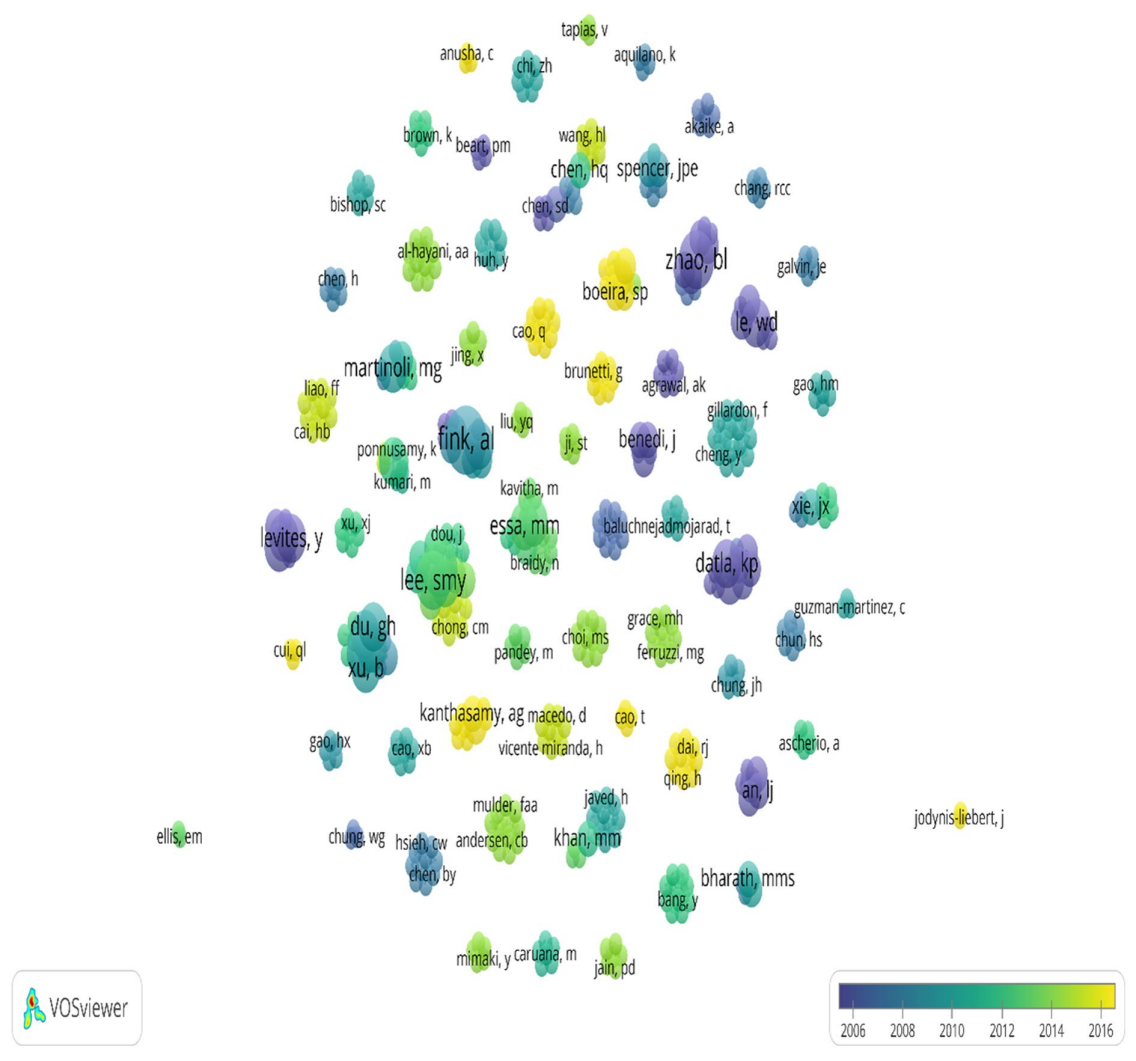
VOSviewer co-authorship map demonstrating the bibliographic coupling between the 497 authors of the 100 most cited articles on PD and PCs. The authors form 66 clusters. The cluster size represents the frequency of publications. The color scale represents the period in which the publications occurred. Overlapping nodes does not allow viewing the name of all authors who contributed to the 100 most cited articles. Authors with the highest number of publications are superimposed on authors with the lowest number of publications.

The top 100 most cited articles were contributed by 497 authors forming 66 clusters (Figure 2). The major contribution with number of paper was made by a Fink, A. L. (*n* = 5) followed by Lee, S. M. Y.; Zhang, Z. J.; Zhao, B. L. (*n* = 4), followed by Datla, K. P.; Dexter, D. T.; Du, G. H.; Essa, M. M.; Haleagrahara, N.; He, G. R.; Le, W. D.; Levites, Y.; Li, G. H.; Li, X. X.; Mandel, S.; Manivasagam, T.; Martinoli, M. G.; Mu, X.; Uversky, V. N.; Xu, B.; Youdim, M. B. H. (*n* = 3). The other authors contributed ≤2 papers.

**Figure 3:**
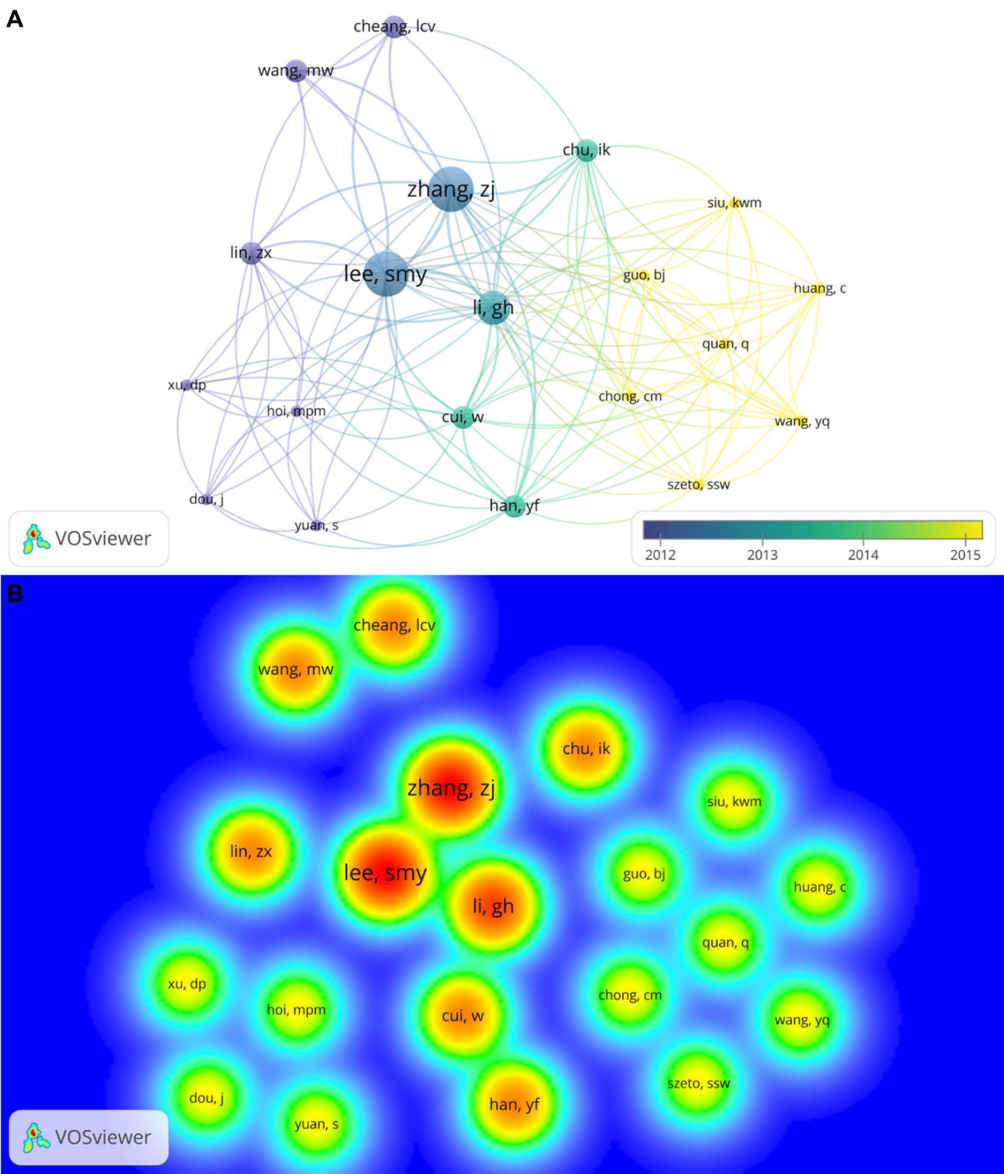
The cluster with the highest number of publications. **(A)** The cluster is composed of 20 authors. The period of publication of the cluster’s papers is indicated by the color scale that varies from blue (2012) to yellow (2015). The node size represents the citation number of each author. The authors who collaborated in publications that occurred between the years 2012–2013 have higher numbers of citations compared to the authors who collaborated with publications that occurred between the years 2014–2015. The number of citations contributes to the strength of the connection between the authors represented by the distance between the authors. Authors with higher numbers of citations have higher binding strength. This representation suggests that the articles published by the cluster in the period 2012–2013 have higher numbers of citations. **(B)** The authors who make up the cluster with the highest number of publications are represented by heat islands demonstrating the citation density (number of citations/year of publication). The size of the heat island corresponds to the citation density. Authors with high citation density are closer, suggesting that publications that occurred in collaboration between them have higher numbers of citations.

The cluster with the most authors is formed by Lee, S. M. Y. and more 19 researchers. Collaborations carried out in 2012 produced more articles, as can be seen by the thickness of the lines between authors who are present in that period of time, for example Lee, S. M.Y. and Zhang, Z. J. Figure 3A in addition, these authors received more citations, as can be seen in the heat map (Figure 3B).

### 3.3. Global distribution of the 100 most cited articles

**Figure 4:**
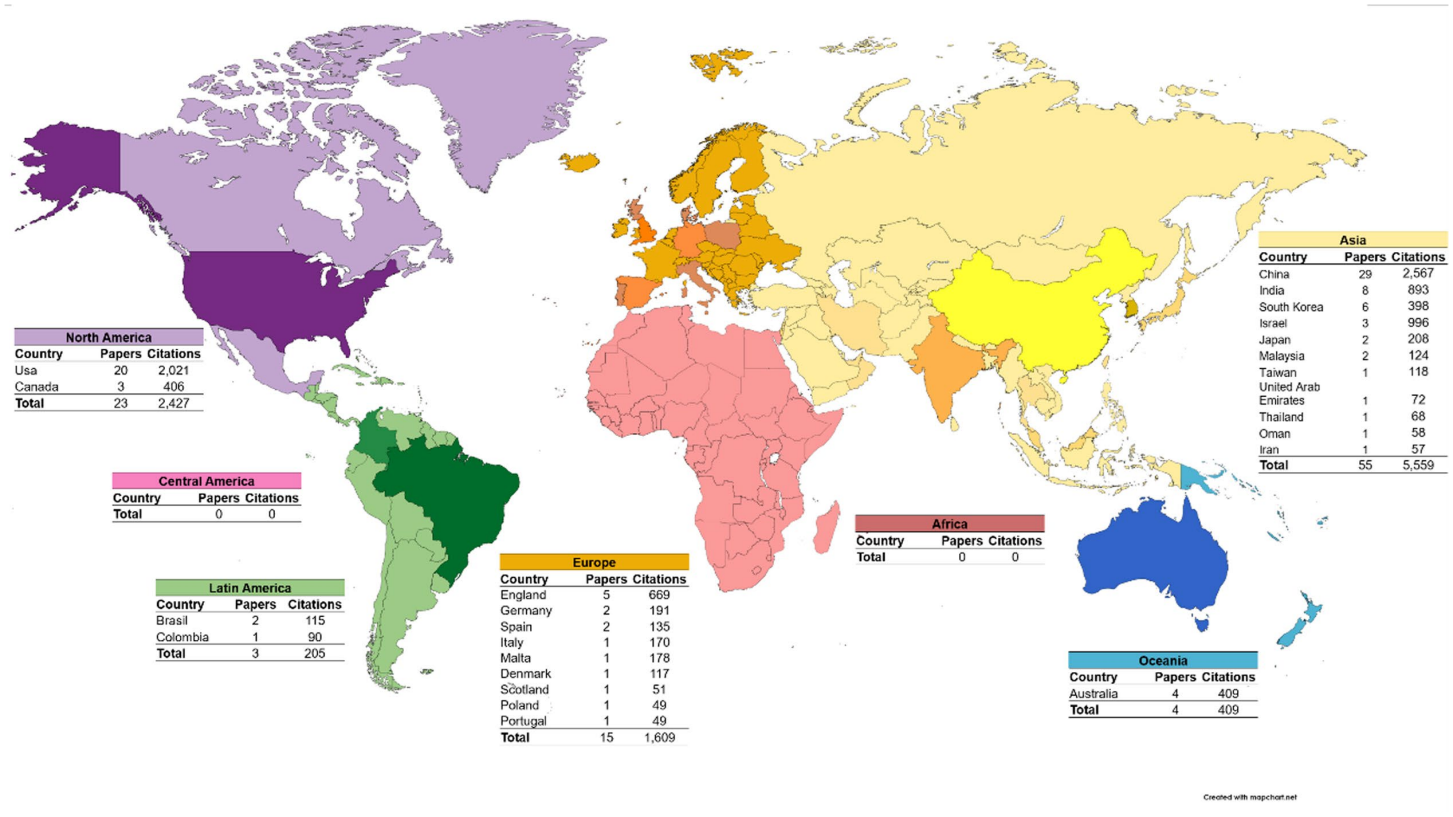
Worldwide distribution of the 100 most cited articles in PD and PC. Countries from the same continent are represented by the same color. The intensity of the color varies according to the presence or absence of publications with a high number of citations on PD and CP by country. Countries with stronger color intensity have publications with a high number of citations on PD and PC.

The list of the 100 most cited articles of WoS-CC has more articles from the Asian (55%) and North America (23%) continents. China is the country with the most articles and most citations on the list. In addition to China, countries like the USA, India and South Korea also have high numbers of articles on the list. The Asian continent has more countries with articles on the list compared to other continents. Africa and Central America are the only continent that does not have countries with articles on the list (Figure 4).

### 3.4. Distribution of the 100 most cited articles by journals, periods, and experimental design

Brain Research Journal has the highest number of articles in the list (5 articles). The journals Free Radical Biology and Medicine and Journal of Neuroscience Research presented four articles. The other journals that are part of the list have less than three articles (Table 3).

**Table 3:** Frequencies of characteristics of the 100 most-cited papers in PD and PC.

Characteristics	*n* papers	*n* citations^a^
Journal (at least 4 papers)		
Brain Research Journal	5	481
Free Radical Biology and Medicine	4	489
Journal of Neuroscience Research	4	462
Period of publications		
2021–2019	1	53
2018–2016	9	526
2015–2013	22	3,681
2012–2010	26	2,249
2009–2007	23	2,479
2006–2004	10	1,505
2003–2001	9	1,554
Study design		
Bibliographic studies	5	563
Observational studies	1	160
Laboratorial studies		
*in vitro + in vivo*	14	1,134
*in vitro + in silico*	1	2,008
*in vivo*	32	3,120
*in vitro*	47	5,124

a Citation count (Web of Science Core Collection).

The most prolific periods in terms of publications on list of the 100 most cited articles of WoS-CC were 2012–2010 (26 publications), 2009–2007 (23 publications), and 2015–2013 (22 publications) respectively (Table 3). On the other hand, the period with most papers citations was 2015–2013 (3,681 citations), followed by 2009–2007 (2,479) and 2012–2010 (2,249).

The most used PD models were *in vitro* (47 papers), *in vivo* (32 papers) and *in vitro* + *in vivo* (14 papers) study designs, totaling the largest number of citations (5,124, 3,120, and 1,134 respectively; Table 3). Other study designs like bibliographic studies that did not have the type of review determined (5 papers), observational study (cross-sectional; 01 paper) and *in vitro* + *in silico* (01 paper) had a low occurrence on the top 100 list in the WoS-CC (Table 3).

### 3.5. Most evaluated compounds, subclasses and therapeutic targets among the 100 most cited articles

The compounds EGCG, quercetin, curcumin e baicalein are the most discussed (19, 15, 9, and 9 papers respectively) in the top 100 list in the WoS-CC, totaling the largest number of citations (2,394, 1,798, 1,183, and 1,060 respectively). The subclasses flavanol, flavones and flavonol are the most discussed (27, 22, and 21 papers respectively) in the top 100 list in the WoS-CC, totaling the largest number of citations (3,354, 2,434, and 2,444, respectively; Table 4).

**Table 4:** Number of articles published by phenolic compounds, subclasses and therapeutic target.

Compound	*n* papers	*n* citations^a^
EGCG	19	2,394
Quercetin	15	1798
Curcumin	9	1,183
Baicalein	9	1,060
Resveratrol	7	981
Epicatechin	5	480
Chrysin	4	397
Genistein	4	379
Catechin	4	323
Naringenin	3	614
Luteolin	3	334
Theaflavin	3	312
Pelargonidin	3	288
Protocatecuic acid	3	260
Caffeic acid	3	259
Gallic acid	3	228
Epigallocatechin	2	200
Tangeritin	2	194
Apigenin	2	184
Epicatechin gallate	2	159
Fraxetin	2	135
Hesperetin	2	131
Myricetin	2	128
Naringin	2	119
Nobiletin	2	106
Subclasses		
Flavanol	27	3,354
Flavone	22	2,434
Flavonol	21	2,444
Stilbene	11	1,455
Curcuminoids	9	1,183
Benzoic acids	9	869
Flavanone	7	976
Isoflavone	7	774
Anthocyanin	5	495
Hydroxycinamic acids	5	542
Proanthocyanidin	4	394
Coumarin	3	186
Lignan	1	178
Xanthone	1	58
Flavanonol	1	56
Therapeutic targets		
Oxidative stress	68	7,183
Neuroinflammation	20	1,821
Fibrils of α-syn	15	1,773
Mitochondrial dysfunction	8	489

a Citation count (Web of Science Core Collection).

The most discussed targeted PD therapy on the WoS-CC Top 100 list was oxidative stress (68 articles), followed by neuroinflammation (20 articles), α-syn fibrils (15 articles) and mitochondrial dysfunction (8 articles; Table 4). However, evaluating the citation ratio it is evident that α-syn fibrils have the highest citation rate per article followed by oxidative stress, neuroinflammation and mitochondrial dysfunction (Table 4).

### 3.6. Keywords that occurred in the 100 most cited articles

**Figure 5:**
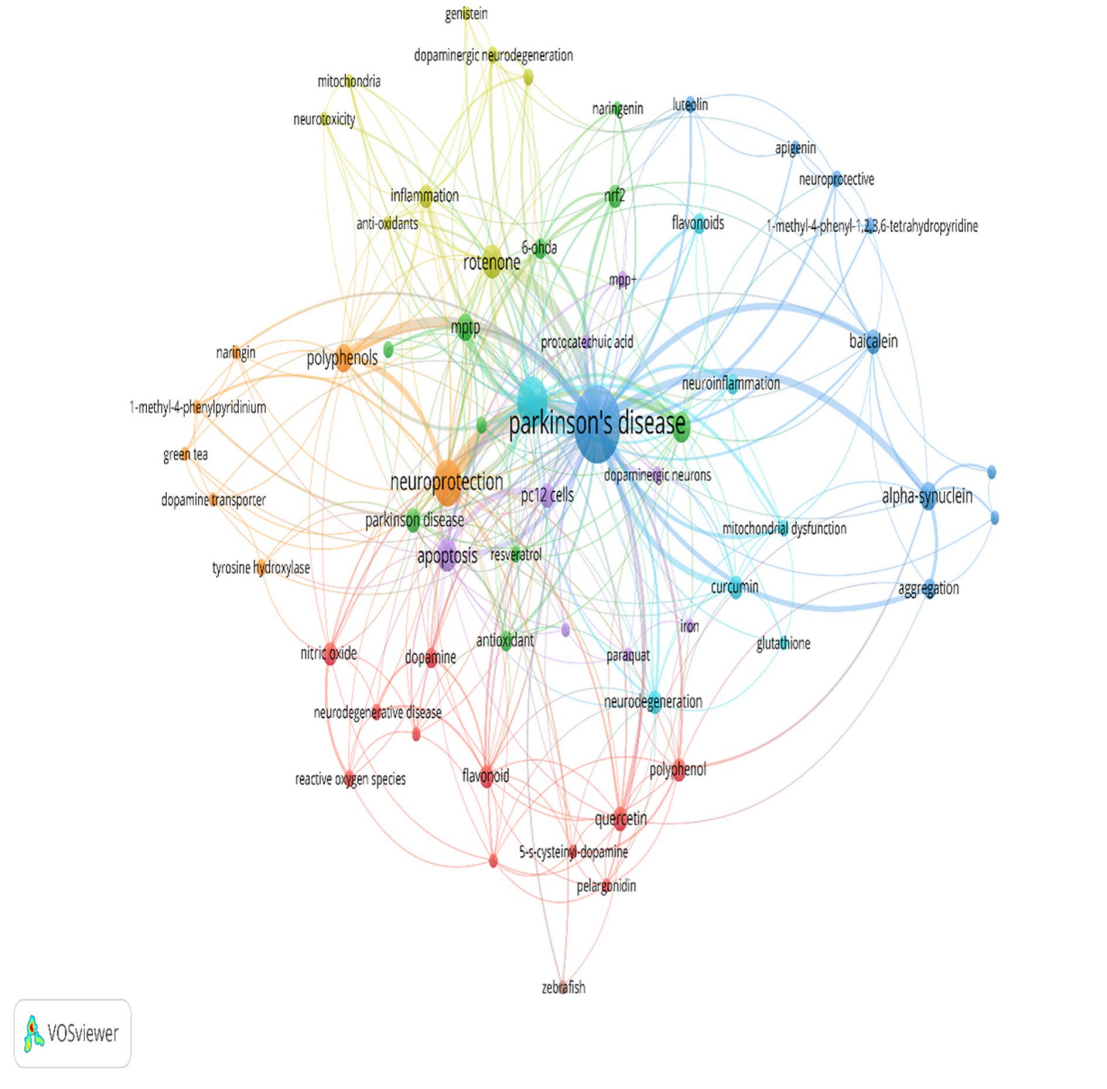
Author keyword network analysis with 2 or more occurrences. The node size represents the frequency of the author keyword with larger nodes indicating greater frequency. The color of the node represents the possibility of co-occurrence of author keywords among the 100 most cited articles on PD and PC with nodes of the same color demonstrating that there is co-occurrence between author keywords in the same article. The thickness of the line between nodes represents the strength of linkage between author keywords with strong linkage strengths being demonstrated by thicker lines. More frequent author keywords have stronger linking strength.

Out of the top 100 most-cited publications, only 82 articles contained author keywords. The keywords that occurred in at least two articles were presented in Figure 5. The most frequently used keyword was parkinson’s disease (*n* = 54), followed by neuroprotection (*n* = 20), apoptosis (*n* = 11), rotenone (*n* = 11), 6-OHDA (*n* = 9), 11 alpha-synuclein (*n* = 8), polyphenols (*n* = 8). A total of 214 author keywords were identified.

## 4. Discussion

From the list of the 100 most cited articles on PD and PCs, the oldest article published in 2001, which also has the highest number of citations and the most recent article published in 2020, was identified. Asia and China were the continent and the country with the highest number of papers on the list. The period with the highest number of articles published was between 2012 and 2010 and the most common experimental design was laboratory studies, especially *in vitro* studies. The compounds EGCG, quercetin, curcumin andbaicalein were the most evaluated compounds and oxidative stress was the most therapeutic target among the papers.

The number of citations indicates the quality of an article according to the scientific community (Fardi et al., 2017). Articles with more than 100 citations are recognized as classics depending on the research area. Classic articles influence research and scientific practice (Arshad et al., 2020). In the list of the 100 most cited, 38 articles received more than 100 citations and can be called classics. The other articles received at least 49 citations.

Older articles tend to accumulate citations over time, then become references, as is the case of the most cited article (*n* citation = 434) in the list that was published in 2001. More recent articles, despite having lower numbers of citations, present new research possibilities within the area of interest (Feijoo et al., 2014). The most recent article on the list demonstrates that the mechanism involved in neuroprotection against PD may also occur due to the anti-inflammatory potential of PCs through the regulation of cytokines and neurotrophic factors (Brunetti et al., 2020).

Author Lee, S. M. Y. leads the cluster with the highest number of research (*n* = 20) in addition to having greater link strength (total link strength = 30), although he is not the author with the highest number of citations (*n* citation = 399). He is a professor and deputy director of the Institute of Chinese Medical Sciences at the University of Macau. He is interested in natural products that can be used as therapeutic agents in brain disorders. His dedication to education and research in the fields of molecular biochemistry biology, pharmacology pharmacy and neuroscience neurology.

PD is the second neurodegenerative disease with the highest incidence in the world. Its prevalence is higher in Anglo-Saxon America and Europe compared to Asia, Latin America and Africa (Benito-León and Louis, 2013). Despite the fact that the Asian continent has a low prevalence of the disease, it was the one that published the most articles on PD and PCs. The research on PD has increased 33-fold in the last 35 years in the Asian continent. This increase may be related to population aging and the new phase of economic development based on the production, communication and consumption of knowledge (Pajo et al., 2020).

China was the country with the most articles and the most citations in the list. In addition to China, countries such as the USA, India and South Korea also had high numbers of articles in the list. The high scientific production on PD and PCs may be related to traditional Chinese medicine (TCM), which has lower costs than Western medicine and more than 170 ingredients for the treatment of the disease, including PCs. The benefits and molecular mechanisms of TCM have been evaluated in preclinical studies, which may lead to the discovery of new therapeutic candidates for the treatment of PD (Li and Le, 2021).

In addition, China already stood out in scientific production on PD between 2013 and 2017, publishing 3,986 articles, behind only the USA. The high number of publications in this period was related to the standardization through guidelines (Chen et al., 2016) on the management of the disease in the country, efforts to reduce the economic burden related to the treatment of the disease and government incentives for publication in English newspapers (Robert et al., 2019).

The number of citations an article receives also contributes to the impact factor that determines the academic prestige of scientific journals. This factor in the year 2020 depended on the sum of the number of citations of early access articles with an early access year in 2020, early access articles with a final publication year in 2020 and an early access year in 2019 or earlier, and unanticipated access articles with a final publication year in 2020 divided by the sum of the number of articles published in the year of evaluation and in the year prior to the evaluation. Thus, journals that have articles with a high number of citations do not necessarily have a high impact factor, as is the case of the 3 three journals with the highest number of articles in the list.

The level of evidence of research is related to the experimental design. According to evidence-based practice systematic reviews and clinical studies are considered more important. As seen, *in vitro* laboratory studies are the most common experimental designs among the 100 articles. The development of these preliminary studies is necessary to carry out an initial screening regarding the effective concentration for bioactivity and toxicity (Ferreira et al., 2017). However, the concentrations evaluated in laboratory studies are not representative of the concentrations and chemical structure that reach the target organ (Perdigão et al., 2023).

After consumption, PCs can be hydrolyzed by intestinal enzymes, but most reach the colon, where they undergo hydrolysis and biotransformation reactions by microbiota enzymes and are then absorbed. Metabolites undergo conjugation reactions in the liver before entering the brain. The penetration of metabolites into the brain depends on the ability to bind to brain efflux transporters present in the blood–brain barrier and on lipophilicity. Metabolites reach the brain at concentrations less than 1 nmol/g of brain tissue (Pandareesh et al., 2015).

More than 10,000 PCs have been identified, of which approximately 500 are dietary compounds (Vuolo et al., 2018). The number of compounds that occurred among the 100 most cited articles (*n* = 51) is not representative of the amount of dietary phenolic compounds, however the compounds EGCG, quercetin, curcumin and baicalein were the most evaluated among the papers (19, 15, 9, and 9 papers, respectively), with the highest number of citations (2,394, 1,798, 1,183, and 1,060, respectively).

EGCG, the most evaluated compound in the list, can be found abundantly in green tea leaves, oolong tea, and black tea leaves (Eng et al., 2018). The estimated daily intake of EGCG through green tea consumption can reach approximately 560 mg/day for individuals consuming an average of 750 ml/day of green tea (Hu et al., 2018). Green tea is among the foods that are prescribed for the prevention of PD in TCM (Li and Le, 2021).

Quercetin, curcumin and baicalein compounds were widely evaluated among the papers as well. Quercetin is mostly conjugated to sugar moieties such as glucose or rutinose and can be found in high concentrations in *Ginkgo Biloba*, a TCM herb, onion, lettuce, chili pepper, cranberry, tomato, broccoli and apple, which contribute to an estimated dietary intake of 6–18 mg/day in the USA, China and the Netherlands (Ishisaka et al., 2011; Guo and Bruno, 2015).

The compound curcumin is extracted from turmeric and is widely used in Asian medicine to treat respiratory and liver diseases and inflammation. The pharmacological activities of curcumin include anti-inflammatory, antimicrobial, antioxidant, among others. Strategies such as nanoencapsulation, liposomes, micelles are being developed to increase bioavailability (Zhenqi et al., 2020).

Baicalein can be found in the root of *Scutellaria baicalensis*, an east Asian plant widely used in TCM to treat diseases (Arredondo et al., 2015). Baicalein has low water solubility for this reason, and it is poorly bioavailable, which makes its application in neuroprotectiv therapies difficult (Zhang et al., 2020). PCs, despite having common structural elements, have structural characteristics such as their degree of oxidation and substituents (position, number and nature of groups attached to rings A and B and the presence of glycosidic bonds) that affect their bioactive potential (Castillo et al., 2000). The subclasses flavanol, flavones, and flavonol were the most discussed (27, 22, and 21 papers, respectively) in the top 100 list in the WoS-CC, with the highest number of citations (3,547, 2,434, and 2,444, respectively; Table 4).

Flavanols, the most evaluated subclass among the 100 articles, present the ortho-dihydroxy (catecholic) group in the B ring, providing the delocalization of electrons, which contributes to a high antioxidant activity, which may be related to the number of studies that evaluated PCs and their effects on oxidative stress as a therapeutic target (Latos-Brozio and Masek, 2019).

The most discussed targeted PD therapy in the WoS-CC top 100 list was oxidative stress (68 articles). The interest in the mechanism used by PCs to reduce intracellular levels of ROS is recent, although the demonstration of the antioxidant potential of PCs in neurons is not (Reglodi et al., 2017). The search for answers makes this therapeutic target the most studied through laboratory models that are important tools, as they provide insights into behavioral improvements in parallel with the improvement in the oxidative state after exposure to PCs, for example, modulating the Nrf-2 signaling pathway and inducing increased expression of antioxidant enzymes such as SOD, CAT, and GSH (Table 2).

Other therapeutic targets for PCs in PD demonstrated in the 100 most cited articles were neuroinflammation (20 articles), α-syn fibrils (15 articles) and mitochondrial dysfunction (8 articles; Table 4). The neuroprotective effects of PCs include reduced expression of cytokines such as IL-6, TNF-α, IL-1b, and COX-2; the breakdown and inhibition of the formation of α-syn fibrils; and the upregulation of complex I activity in the mitochondria (Table 2).

Keywords are essential for discovering scientific articles. They are used as codes to access literature in a particular area. When using keywords in the search, you get more relevant results compared to using a phrase. Despite its importance, there are articles that do not bring keywords and make retrieval difficult during the search (Natarajan et al., 2010).

The most used keywords among the 100 articles include neuroprotection 6-OHDA antioxidant apoptosis. These words can help in the search for articles related to the topic in addition to indicating an overview of the research because they are words that represent therapeutic targets mechanisms of the neuroprotective effect and neurotoxins used in the papers.

## 5. Conclusion

The present study identified the 100 most cited articles on PD and PCs. The increased incidence of aging-related diseases due to the increase in the number of elderly people in the world has motivated countries such as China and the USA to seek other strategies for the treatment of PD in order to reduce the side effects and costs of available treatment. Plant foods and beverages have been used for a long time by TCM to treat neurodegenerative diseases, encouraging the search for the mechanisms behind the neuroprotective effect. Research mainly in laboratory models on the use of PCs against PD has grown since 2007 and has highlighted bioactive potentials that include antioxidant and anti-inflammatory activity. Despite the promising results obtained, clinical studies are needed to obtain more conclusive answers about the neuroprotective effects of PCs in humans, as the bioactive potential is influenced by bioavailability.

## Author contributions

JP, BT, and DB-d-S performed experiments, analyzed and interpreted the data, and drafted the manuscript. PN, RL, and HR formulated the study concept, designed the study, and made critical revisions of the manuscript. All authors contributed to the article and approved the submitted version.

## Conflict of interest

The authors declare that the research was conducted in the absence of any commercial or financial relationships that could be construed as a potential conflict of interest.

## Publisher’s note

All claims expressed in this article are solely those of the authors and do not necessarily represent those of their affiliated organizations, or those of the publisher, the editors and the reviewers. Any product that may be evaluated in this article, or claim that may be made by its manufacturer, is not guaranteed or endorsed by the publisher.

## Figures and Tables

**Table:** 

5-HT	Serotonin
6-OHDA	6-Hydroxydopamine
α-syn	α-synuclein
AChE	Acetylcholinesterase
Akb	Protein kinase B
ARE	Antioxidant response elemento
Bax	BCL2 Associated X
BBB	Blood–brain barrier
BCL2	BCL2 Apoptosis Regulator
BDNF	Brain-derived neurotrophic fator
BTE	Black tea extract
CaMKII	Ca^2+^/calmodulin-dependent protein kinase II
CAT	Catalase
C-Jun	Protein encoded by the JUN gene
COMT	Catechol O-methyltransferase
Cyt c	Cytochrome c
DA	Dopamine
DARPP-32	Dopamine- and cAMP-regulated phosphoprotein-32
EGCG	Epigallocatechin gallate
ERK 1/2	Extracellular signal-regulated kinases
GDNF	Glial cell-derived neurotrophic fator
GPx	Glutathione peroxidase
GSH	Glutathione
GSK-3β	Glycogen synthase kinase-3β
GTP	Green tea polyphenols
HMOX1	Heme oxygenase-1
HVA	Homovanillic acid
IFN-γ	Interferon-gamma
IL	Interleukin
iNOS	Nitric oxide synthase
JNK 1/2	c-Jun N-terminal cinase
Keap1	Kelch-like ECH-associated protein 1
LDH	Lactate dehydrogenase
MAO-Bis	monoamine oxidase type B inhibitors
MDA	Malondialdehyde
MMP	Mitochondrial Membrane Potential
mRNA	Messenger ribonucleic acid
NDGA	Nordihydroguaiaretic acid
NF-ĸB	Nuclear factor-kappa B
NO	Nitric oxide
NQO1	NAD(P)H:quinone oxidoreductase
Nrf-2	Erythroid nuclear factor-2 related to factor 2
P38	p38 mitogen-activated protein kinases
PCa	Carbonyl protein
PC	Phenolic compound
PD	Parkinson’s disease
PIK-3	Phosphoinositide 3-kinase
PKC	Protein kinase C
PLA2	Phospholipase A2
ROS	Reactive oxygen species
SMAC	Second mitochondria-derived activator of caspase
SOD	Superoxide dismutase
TBARS	Thiobarbituric acid reactive substances
TCM	Traditional Chinese medicine
TH	Tyrosine hydroxylase
TH-ir	TH-immunoreactive
TNF-α	Tumor necrosis factor alpha
TRAP	Total reactive antioxidant potential
VOSviewer	Visualization of Similarities Viewer
WoS-CC	Web of Science Core Collection
