# Supplementary material for: Analysis of phenolic compounds in Parkinson’s disease: a bibliometric assessment of the 100 most cited papers
Source: Front Aging Neurosci. 2023 May 2;15:1149143. doi: 10.3389/fnagi.2023.1149143 (PMC10185771; doi:10.3389/fnagi.2023.1149143)
Supplement: Supplementary file 1 [file Table_1.pdf]

ANALYSIS OF PHENOLIC COMPOUNDS IN PARKINSON'S DISEASE: A BIBLIOMETRIC ASSESSMENT OF THE 100 MOST CITED PAPERS

José Messias Perdigão[a], Bruno José Brito Teixeira[a], Daiane Claydes Baia-da-Silva[b], Priscila Cunha Nascimento[b], Rafael Rodrigues Lima[b], Hervé Rogez[a*]

[a]Centre for Valorization of Amazonian Bioactive Compounds, Federal University of Pará, Belém, PA, Brazil
[b]Laboratory of Functional and Structural Biology, Institute of Biological Sciences, Federal University of Pará, Belém, PA, Brazil

*Corresponding author at: Centre for Valorization of Amazonian Bioactive Compounds (CVACBA), Federal University of Pará, Av. Perimetral da Ciência, km 01, Belém 66075-750, Brazil. E-mail address: frutas@ufpa.br (H. Rogez).

This Supplementary Material contains one table

Supplementaru Table 1: Excluded papers with reason

Citation number in WoS-CC	Paper	Authors	Reasons
**1,594**	Advances in metal-induced oxidative stress and human disease	Jomova, K. and Valko, M.	The article doesn’t investigate phenolic compounds
**1,048**	Role of free radicals in the neurodegenerative diseases - Therapeutic implications for antioxidant treatment	Halliwell, B.	The article investigate applications of several antioxidant compounds in neurodegenerative diseases, not specifically Parkinson's disease
**951**	EGCG redirects amyloidogenic polypeptides into unstructured, off-pathway oligomers	Ehrnhoefer et al.	The article studies inhibition of β-amyloid fibrils associated with other neurodegenerative diseases, not specifically Parkinson's disease.
**774**	Multi-target-directed ligands to combat neurodegenerative diseases	Cavalli et al.	The compound evaluated in that article isn’t a phenolic compound
**741**	Free radicals and grape seed proanthocyanidin extract: importance in human health and disease prevention	Bagchi et al.	The experiments tested in the article are nonspecific to indicate outcomes related only to Parkinson's disease
**732**	Inhibition of amyloid fibril formation by polyphenols: Structural similarity and	Porat, Y.; Abramowitz, A. and Gazit, E.	The article studies inhibition of β-amyloid fibrils relacionted with other neurodegenerative diseases, not specifically Parkinson's disease

	aromatic interactions as a common inhibition mechanism		
**686**	Metals, oxidative stress and neurodegenerative disorders	Jomova et al.	The article investigates the oxidative stress caused by metals in Parkinson's and Alzheimer's diseases and the role of antioxidants in the development of both diseases, not specifically Parkinson's disease
**635**	EGCG remodels mature alpha-synuclein and amyloid-beta fibrils and reduces cellular toxicity	Bieschke et al.	The article studies inhibition of β-amyloid fibrils relacionted with other neurodegenerative diseases, not specifically Parkinson's disease
**627**	A Review of the Antioxidant Mechanisms of Polyphenol Compounds Related to Iron Binding	Perron, R. and Brumaghim, J.	The article explains the conditions that the iron íon can generate oxidative stress and cause Parkinson, but didn't investigate the neuroprotective effect of phenolic compounds in Parkinson's disease
**618**	Flavonoids protect neuronal cells from oxidative stress by three distinct mechanisms	Ishige, K.; Schubert, D. and Sagara, Y.	The article didn't investigate the neuroprotective effect of phenolic compounds in Parkinson's disease, only mentions that oxidative stress can generate Parkinson
**532**	DJ-1, a cancer- and Parkinson's disease-associated protein, stabilizes the antioxidant transcriptional master regulator Nrf2	Clements et al.	The article is not about phenolic compounds and doesn't have the word phenolic compound or synonyms. The article investigate applications in neurodegenerative diseases, not specifically Parkinson's disease
**512**	Cyclooxygenase-2 is instrumental in Parkinson's disease neurodegeneration	Teismann et al.	The compound evaluated in that article isn't a phenolic compound
**511**	Dopamine oxidation alters mitochondrial respiration and	Berman, S. B. and Hastings, T. G.	The compound evaluated in that article isn't a phenolic compound

	induces permeability transition in brain mitochondria: Implications for Parkinson's disease		
**475**	Emerging role of polyphenolic compounds in the treatment of neurodegenerative diseases: A review of their intracellular targets	Ramassamy, C	The article investigate applications of polyphenolic compounds in neurodegenerative diseases, not specifically Parkinson's disease
**473**	Antioxidant activity and biologic properties of a procyanidin-rich extract from pine (Pinus maritima) bark, pycnogenol	Packer, L.; Rimbach, G. and Virgili, F.	The experiments tested in the article are nonspecific to indicate outcomes related only to Parkinson's disease
**442**	Oxidative stress, nutrition and health. Experimental strategies for optimization of nutritional antioxidant intake in humans	Halliwell, B.	The article doesn’t investigate Parkinson's disease
**433**	Role of oxidation in the neurotoxic effects of intrastriatal dopamine injections	Hastings, T. G.; Lewis, D. A. and Zigmond, M. J.	The article doesn’t investigate phenolic compounds
**430**	Physiological effects of melatonin: Role of melatonin receptors and signal transduction pathways	Pandi-Perumal et al.	The article doesn’t investigate Parkinson's disease and phenolic compounds
**420**	Effects of dietary flavonoids on apoptotic pathways related to cancer chemoprevention	Ramos, S.	The experiments tested in the article are nonspecific to indicate outcomes related only to Parkinson's disease

**409**	A novel neurological phenotype in mice lacking mitochondrial manganese superoxide dismutase	Melov et al.	The article doesn’t investigate Parkinson's disease and phenolic compounds
**408**	Nrf2-mediated neuroprotection in the MPTP mouse model of Parkinson's disease: Critical role for the astrocyte	Chen et al.	The compounds evaluated in that article isn’t a phenolic compounds
**391**	Inhibition of heparin-induced tau filament formation by phenothiazines, polyphenols, and porphyrins	Taniguchi et al.	The experiments tested in the article are nonspecific to indicate outcomes related only to Parkinson's disease
**386**	Redox- and non-redox-metal-induced formation of free radicals and their role in human disease	Valko et al.	The article investigate applications of antioxidants compounds in neurodegenerative diseases, not specifically Parkinson's disease
**381**	Dopamine- or L-DOPA-induced neurotoxicity: The role of dopamine quinone formation and tyrosinase in a model of Parkinson's disease	Asanuma, M.; Miyazaki, I. and Ogawa, N.	The article doesn’t investigate phenolic compounds
**374**	The thioflavin T fluorescence assay for amyloid fibril detection can be biased by the presence of exogenous compounds	Hudson et al.	The experiments tested in the article are nonspecific to indicate outcomes related only to Parkinson's disease
**356**	Exercise and the brain: something to chew on	Van Praag, H.	The article doesn’t investigate Parkinson's disease and phenolic compounds

**329**	Dietary supplementation with resveratrol reduces plaque pathology in a transgenic model of Alzheimer's disease	Karuppagounder, et al.	The experiments tested in the article are nonspecific to indicate outcomes related only to Parkinson's disease
**316**	Neurological mechanisms of green tea polyphenols in Alzheimer's and Parkinson's diseases	Weinreb et al.	The article investigates applications of polyphenolic compounds in Alzheimer's and Parkinson's diseases, not specifically for Parkinson's
**315**	Enzymatic oxidation of dopamine - the role of prostaglandin-h synthase	Hastings, T. G.	The article doesn’t investigate phenolic compounds
**301**	Catechin polyphenols: Neurodegeneration and neuroprotection in neurodegenerative diseases	Mandel, S. and Youdim, M. B. H.	The article investigate applications of phenolic compounds in neurodegenerative diseases, not specifically Parkinson's disease
**289**	Conjugates of catecholamines with cysteine and GSH in Parkinson's disease: Possible mechanisms of formation involving reactive oxygen species	Spencer et al.	The article doesn’t investigate phenolic compounds
**288**	The iron chelator desferrioxamine (desferal) retards 6-hydroxydopamine-induced degeneration of nigrostriatal dopamine neurons	Benshachar et al.	The compound evaluated in that article isn’t a phenolic compound
**283**	Neuromelanin biosynthesis is driven by excess cytosolic catecholamines not	Sulzer et al.	The compound evaluated in that article isn’t a phenolic compound

	accumulated by synaptic vesicles		
**278**	Neuroprotection and neurorescue against A beta toxicity and PKC-dependent release of non-amyloidogenic soluble precursor protein by green tea polyphenol (-)-epigallocatechin-3-gallate	Levites et al.	This article is not related to a model of Parkinson's disease development
**276**	Cell signaling pathways in the neuroprotective actions of the green tea polyphenol (-)-epigallocatechin-3-gallate: implications for neurodegenerative diseases	Mandel et al.	The article investigate applications of phenolic compounds in neurodegenerative diseases, not specifically Parkinson's disease
**271**	Lactoferrin-conjugated PEG-PLA nanoparticles with improved brain delivery: In vitro and in vivo evaluations	Hu et al.	This article is not related to a model of Parkinson's disease development
**263**	Glutathione transferases catalyse the detoxication of oxidized metabolites (o-quinones) of catecholamines and may serve as an antioxidant system preventing degenerative cellular processes	Baez et al.	The compound evaluated in that article isn't a phenolic compound
**259**	Towards a unifying, systems biology understanding of large-scale cellular death and destruction caused by poorly	Kell, D. B.	The article didn't investigate the neuroprotective effect of phenolic compounds in Parkinson's disease

	liganded iron: Parkinson's, Huntington's, Alzheimer's, prions, bactericides, chemical toxicology and others as examples		
**258**	Small molecule inhibitors of alpha-synuclein filament assembly	Masuda et al.	The article investigate applications of several antioxidant compounds, not specifically phenolic compounds
**258**	Multifunctional activities of green tea catechins in neuroprotection - Modulation of cell survival genes, iron-dependent oxidative stress and PKC signaling pathway	Mandel et al.	The article investigate applications of phenolic compounds in neurodegenerative diseases, not specifically Parkinson's disease
**252**	Ascorbic-acid protects against levodopa-induced neurotoxicity on a catecholamine-rich human neuroblastoma cell-line	Pardo et al.	The compound evaluated in that article isn't a phenolic compound
**248**	The beneficial role of curcumin on inflammation; diabetes and neurodegenerative disease: A recent update	Ghosh, S.; Banerjee, S. and Sil, P. C.	The article investigate applications of phenolic compound in neurodegenerative diseases, not specifically Parkinson's disease
**248**	Coffee: biochemistry and potential impact on health	Ludwig et al.	The article doesn't investigate Parkinson's disease and phenolic compounds
**240**	Autoxidation and neurotoxicity of 6-hydroxydopamine in the presence of some antioxidants: Potential implication in relation	Soto-Otero et al.	The compound evaluated in that article isn't a phenolic compound

	to the pathogenesis of Parkinson's disease		
**229**	Naturally occurring phytochemicals for the prevention of Alzheimer's disease	Kim, J.; Lee, H. J.; Lee, K. W.	The article doesn’t investigate Parkinson's disease
**226**	Two distinct mechanisms are involved in 6-hydroxydopamine- and MPP+-induced dopaminergic neuronal cell death: Role of caspases, ROS, and JNK	Choi et al.	The article doesn’t investigate phenolic compounds
**225**	Skin whitening agents: medicinal chemistry perspective of tyrosinase inhibitors	Pillaiyar, T.; Manickam, M. and Namasivayam, V.	The article doesn’t investigate Parkinson's disease and phenolic compounds specifically
**225**	A review of specific dietary antioxidants and the effects on biochemical mechanisms related to neurodegenerative processes	Esposito et al.	The article doesn’t investigate Parkinson's disease and phenolic compounds specifically
**222**	Toward the Molecular Mechanism(s) by Which EGCG Treatment Remodels Mature Amyloid Fibrils	Palhano et al.	This article isn’t related to a model of Parkinson’s disease development
**220**	Antioxidant compounds have potent anti-fibrillogenic and fibril-destabilizing effects for alpha-synuclein fibrils in vitro	Ono, K. and Yamada, M.	The article doesn’t investigate Parkinson's disease and phenolic compounds specifically

**217**	Curcumin and its Derivatives: Their Application in Neuropharmacology and Neuroscience in the 21st Century	Lee et al.	The article investigate applications of phenolic compound in neurodegenerative diseases, not specifically Parkinson's disease
**217**	Role of oxidative stress and antioxidants in neurodegenerative diseases	Rao, A. V.; Balachandran, B.	The article investigate applications of antioxidant compounds in neurodegenerative diseases, not specifically Parkinson's disease
**216**	Microglial activation and dopaminergic cell injury: An in vitro model relevant to Parkinson's disease	Le et al.	The article doesn’t investigate phenolic compounds
**207**	Resveratrol as a Therapeutic Agent for Neurodegenerative Diseases	Sun et al.	The article investigate applications of phenolic compound in neurodegenerative diseases, not specifically Parkinson's disease
**203**	Protective and toxic roles of dopamine in Parkinson's disease	Segura-Aguilar et al.	The article doesn’t investigate Parkinson's disease
**203**	Green tea catechins as brain-permeable, natural iron chelators-antioxidants for the treatment of neurodegenerative disorders	Mandel et al.	The article investigate applications of phenolic compounds in neurodegenerative diseases, not specifically Parkinson's disease
**201**	Myricetin: A Dietary Molecule with Diverse Biological Activities	Semwal et al.	The article investigate applications of phenolic compound in neurodegenerative diseases, not specifically Parkinson's disease
**199**	Antioxidant properties of natural polyphenols and their therapeutic potentials for Alzheimer's disease	Choi et al.	This article isn’t related to a model of Parkinson’s disease development

**199**	Resveratrol inhibits beta-amyloid oligomeric cytotoxicity but does not prevent oligomer formation	Feng et al.	This article isn’t related to a model of Parkinson’s disease development
**197**	The Flavanol (-)-Epigallocatechin 3-Gallate Inhibits Amyloid Formation by Islet Amyloid Polypeptide, Disaggregates Amyloid Fibrils, and Protects Cultured Cells against IAPP-Induced Toxicity	Meng et al.	This article isn’t related to a model of Parkinson’s disease development
**197**	Intraneuronal dopamine-quinone synthesis: A review	Sulzer, D. and Zecca, L.	The article doesn’t investigate phenolic compounds
**190**	Flavonoids and the CNS	Jager, A. K. and Saaby, L.	The article investigate applications of phenolic compound in neurodegenerative diseases, not specifically Parkinson's disease
**187**	Pharmacological Targeting of the Transcription Factor Nrf2 at the Basal Ganglia Provides Disease Modifying Therapy for Experimental Parkinsonism	Jazwa et al.	The article doesn’t investigate phenolic compounds
**185**	Polyphenols: Multipotent Therapeutic Agents in Neurodegenerative Diseases	Bhullar, K. S. and Rupasinghe, H. P. V.	The article investigate applications of phenolic compound in neurodegenerative diseases, not specifically Parkinson's disease
**185**	Anti-inflammatory activities of resveratrol in the brain: Role of resveratrol in microglial activation	Zhang, F.; Liu, J. nad Shi, J. S.	The article investigate applications of phenolic compound in neurodegenerative diseases, not specifically Parkinson's disease

**183**	Kinetic and structural analysis of the early oxidation products of dopamine - Analysis of the interactions with alpha-synuclein	Bisaglia, M.; Mammi, S. and Bubacco, L.	The article doesn’t investigate phenolic compounds
**183**	Involvement of cannabinoid receptors in the regulation of neurotransmitter release in the rodent striatum: A combined immunochemical and pharmacological analysis	Kofalvi et al.	The article doesn’t investigate Parkinson's disease and phenolic compounds
**178**	Neuroinflammation: Modulation by flavonoids and mechanisms of action	Spencer et al.	The article investigate applications of phenolic compound in neuroinflmation, not specifically Parkinson's disease
**178**	Dopamine and L-dopa disaggregate amyloid fibrils: implications for Parkinson's and Alzheimer's disease	Li et al.	The article doesn’t investigate phenolic compounds and investigate disaggregate amyloid fibrils in neurodegenerative diseases, not specifically Parkinson's disease
**177**	Tyrosinase mRNA is expressed in human substantia nigra	Xu et al.	The article doesn’t investigate phenolic compounds
**173**	Imbalance in antioxidant defence and human diseases: Multiple approach of natural antioxidants therapy	Tiwari, A. K.	The article investigate applications of several antioxidant compounds, not specifically phenolic compounds, in neurodegenerative diseases, not specifically Parkinson's disease
**172**	Nrf2-a therapeutic target for the treatment of neurodegenerative diseases	Johnson, D. A. and Johnson, J. A.	The article doesn’t investigate phenolic compounds
**172**	Natural polyphenols against neurodegenerative disorders: Potentials and pitfalls	Ebrahimi, A. and Schluesener, H.	The article investigate applications of phenolic compounds in neurodegenerative diseases, not specifically Parkinson's disease

**169**	Novel multifunctional neuroprotective iron chelator-monoamine oxidase inhibitor drugs for neurodegenerative diseases: in vitro studies on antioxidant activity, prevention of lipid peroxide formation and monoamine oxidase inhibition	Zheng et al.	The article investigate applications of synthetic compounds, not specifically phenolic compounds, in neurodegenerative diseases, not specifically Parkinson's disease
**169**	Biochemical and Therapeutic Effects of Antioxidants in the Treatment of Alzheimer's Disease, Parkinson's Disease, and Amyotrophic Lateral Sclerosis	Di Matteo, V. and Esposito, E.	The article investigate applications of antioxidant compounds, not specifically phenolic compounds, in neurodegenerative diseases, not specifically Parkinson's disease
**165**	Luteolin as an anti-inflammatory and neuroprotective agent: A brief review	Nabavi et al.	The article investigate applications of phenolic compound in neuroprotection, not specifically Parkinson's disease
**164**	Catechol ortho-quinones: the electrophilic compounds that form depurinating DNA adducts and could initiate cancer and other diseases	Cavalieri et al.	The article doesn’t investigate Parkinson's disease and phenolic compounds
**161**	Effects of natural antioxidants in neurodegenerative disease	Luz et al.	The article investigate applications of phenolic compounds in neurodegenerative diseases, not specifically Parkinson's disease
**161**	The herbicide paraquat induces dopaminergic nigral apoptosis through sustained activation of the JNK pathway	Peng et al.	The article doesn’t investigate phenolic compounds

**160**	Traditional usages, botany, phytochemistry, pharmacology and toxicology of Polygonum multiflorum Thunb.: A review	Lin et al.	The article investigate applications of several compounds, not specifically phenolic compounds and not specifically applicate in Parkinson's disease
**159**	Clinical aspects of coenzyme Q(10): An update	Littarru, G. P.; Tiano, L.	The article doesn't investigate phenolic compounds, only Coenzyme Q10 in neurodegenerative diseases, not specifically Parkinson's disease
**154**	Health benefits of anthocyanins and molecular mechanisms: Update from recent decade	Li et al.	The article investigate applications of phenolic compounds in molecular mechanisms, not specifically Parkinson's disease
**154**	The beneficial effects of fruit polyphenols on brain aging	Lau, F. C.; Shukitt-Hale, B. and Joseph, J. A.	The article investigate applications of phenolic compounds in brain, not specifically Parkinson's disease
**153**	Neuroprotective Effect of Natural Products Against Alzheimer's Disease	Essa et al.	The article doesn't investigate Parkinson's disease
**153**	Neuroprotective molecular mechanisms of (-)-epigallocatechin-3-gallate: a reflective outcome of its antioxidant, iron chelating and neuritogenic properties	Weinreb et al.	The article investigate applications of phenolic compound in neuritogenic properties, not specifically Parkinson's disease
**152**	Inhibiting toxic aggregation of amyloidogenic proteins: A therapeutic strategy for protein misfolding diseases	Cheng et al.	The article investigate applications of several compounds, not specifically phenolic compounds in protein misfolding diseases, not specifically Parkinson's disease
**152**	Therapeutic approaches to inflammation in neurodegenerative disease	Klegeris, A.; McGeer, E. G. and McGeer, P. L.	The article investigate applications of several compounds, not specifically phenolic

			compounds in neurodegenerative diseases, not specifically Parkinson's disease
**152**	Dietary antioxidants and Parkinson disease - The Rotterdam study	De Rijk et al.	The article investigate applications of several antioxidants compounds, not specifically phenolic compounds
**151**	Lactoferrin conjugated PEG-PLGA nanoparticles for brain delivery: Preparation, characterization and efficacy in Parkinson's disease	Hu et al.	The article investigate applications of synthetic compounds, not specifically phenolic compounds
**148**	Effects of Grape Seed-derived Polyphenols on Amyloid beta-Protein Self-assembly and Cytotoxicity	Ono et al.	The article doesn't investigate Parkinson's disease
**147**	A mitochondrial superoxide theory for oxidative stress diseases and aging	Indo et al.	The article doesn't investigate Parkinson's disease and phenolic compounds
**147**	Phenolic antioxidants attenuate neuronal cell death following uptake of oxidized low-density lipoprotein	Schroeter et al.	This article is not related to a model of Parkinson's disease development
**146**	Botanical Phenolics and Brain Health	Sun et al.	The article investigate applications of several compounds, not specifically phenolic compounds in brain health, not specifically Parkinson's disease
**145**	Identification of catechol-protein conjugates in neostriatal slices incubated with [h-3] dopamine - impact	Hastings, T. G. and Zigmond, M. J.	The article doesn't investigate phenolic compounds

	of ascorbic-acid and glutathione		
**144**	Modification of dopamine transporter function: Effect of reactive oxygen species and dopamine	Berman, S. B.; Zigmond, M. J. and Hastings, T. G.	The article doesn’t investigate phenolic compounds
**143**	Involvement of Astrocytes in Alzheimer's Disease from a Neuroinflammatory and Oxidative Stress Perspective	Gonzalez-Reyes et al.	The article doesn’t investigate Parkinson's disease
**143**	Berry fruit supplementation and the aging brain	Shukitt-Hale, B.; Lau, F. C. and Joseph, J. A.	The article investigate applications of several compounds, not specifically phenolic compounds in brain health, not specifically Parkinson's disease
**143**	Simultaneous Manipulation of Multiple Brain Targets by Green Tea Catechins: A Potential Neuroprotective Strategy for Alzheimer and Parkinson Diseases	Mandel et al.	The article investigate applications of phenolic compounds in Alzheimer and Parkinson Diseases, not specifically Parkinson's disease
**142**	Phenolic Compounds Prevent Amyloid beta-Protein Oligomerization and Synaptic Dysfunction by Site-specific Binding	Ono et al.	The article doesn’t investigate Parkinson's disease
**142**	Is coffee a functional food?	Dorea, J. G. and da Costa, T. H. M.	The article doesn’t investigate Parkinson's disease and phenolic compounds specifically
**140**	Tyrosine hydroxylase is inactivated by catechol-quinones and converted to a	Kuhn et al.	The article doesn’t investigate phenolic compounds

	redox-cycling quinoprotein: Possible relevance to Parkinson's disease		
**138**	Annonacin, a natural mitochondrial complex I inhibitor, causes tau pathology in cultured neurons	Escobar-Khondiker et al.	The article doesn’t investigate phenolic compounds and isn’t related to a model of Parkinson’s disease development
**138**	Dopamine, in the presence of tyrosinase, covalently modifies and inactivates tyrosine hydroxylase	Xu et al.	The article doesn’t investigate phenolic compounds
**135**	alpha-Synuclein expression and Nrf2 deficiency cooperate to aggravate protein aggregation, neuronal death and inflammation in early-stage Parkinson's disease	Lastres-Becker et al.	The article doesn’t investigate phenolic compounds
**134**	Structural Properties of Pore-Forming Oligomers of alpha-Synuclein	Kim et al.	The article doesn’t investigate phenolic compounds. Study oligomers of α-Synuclein, not specifically Parkinson's disease
**133**	Effects of l-cysteine on the oxidation chemistry of dopamine - new reaction pathways of potential relevance to idiopathic parkinsons-disease	Zhang, F. and Dryhurst, G.	The article doesn’t investigate phenolic compounds
**131**	Modulation of neurotrophic signaling pathways by polyphenols	Moosavi et al.	The article investigate modulation in neurotrophic signaling pathways by phenolic compounds, not specifically in Parkinson's disease

**131**	Quercetin and related polyphenols: new insights and implications for their bioactivity and bioavailability	Kawabata, K.; Mukai, R.; Ishisaka, A.	The article doesn’t investigate Parkinson's disease and phenolic compounds specifically
**131**	Morus alba L. nature's functional tonic	Butt et al.	The article doesn’t investigate Parkinson's disease and phenolic compounds specifically
**131**	Molecular mechanisms of 6-hydroxydopamine-induced cytotoxicity in PC12 cells: Involvement of hydrogen peroxide-dependent and -independent action	Saito et al.	The article doesn’t investigate phenolic compounds
**131**	Dieldrin induces apoptosis by promoting caspase-3-dependent proteolytic cleavage of protein kinase C delta in dopaminergic cells: Relevance to oxidative stress and dopaminergic degeneration	Kitazawa, M.; Anantharam, V. and Kanthasamy, A. G.	The article doesn’t investigate phenolic compounds
**130**	Anti-inflammatory effects of flavonoids in neurodegenerative disorders	Spagnuolo, C.; Moccia, S. and Russo, G. L.	The article investigate applications of phenolic compounds in neurodegenerative disorders, not specifically Parkinson's disease
**130**	Oxidative damage and the Nrf2-ARE pathway in neurodegenerative diseases	Gan, L. and Johnson, J. A.	The article doesn’t investigate phenolic compounds. Study several neurodegenerative diseases, not specifically Parkinson's disease
**130**	The role of dopamine oxidation in mitochondrial dysfunction: implications for Parkinson's disease	Hastings, T. G.	The article doesn’t investigate phenolic compounds

**130**	Neuromelanin can protect against iron-mediated oxidative damage in system modeling iron overload of brain aging and Parkinson's disease	Zecca et al.	The article doesn’t investigate phenolic compounds
**128**	Manganese-induced neurotoxicity: a review of its behavioral consequences and neuroprotective strategies	Peres et al.	The article investigate applications of several antioxidant compounds, no specifically phenolic compounds, in neuroprotection, not specifically Parkinson's disease
**128**	Rutin improves spatial memory in Alzheimer's disease transgenic mice by reducing A beta oligomer level and attenuating oxidative stress and neuroinflammation	Xu et al.	This article is not related to a model of Parkinson’s disease development
**127**	Aging: An important factor for the pathogenesis of neurodegenerative diseases	Farooqui, T. and Farooqui, A. A.	The article investigate the aging factor and doesn’t investigate phenolic compounds. Study neurodegenerative diseases, not specifically Parkinson's disease
**127**	Iron dysregulation in Alzheimer's disease: Multimodal brain permeable iron chelating drugs, possessing neuroprotective-neurorescue and amyloid precursor protein-processing regulatory activities as therapeutic agents	Mandel et al.	This article is not related to a model of Parkinson’s disease development

**126**	Coumarin: A Natural, Privileged and Versatile Scaffold for Bioactive Compounds	Stefanachi et al.	The article investigate applications of phenolic compound in neurodegenerative diseases, not specifically Parkinson's disease
**125**	Protein Folding and Aggregation into Amyloid: The Interference by Natural Phenolic Compounds	Stefani, M. and Rigacci, S.	The article doesn’t investigate Parkinson's disease specifically
**125**	Suppressive effects of flavonoid fisetin on lipopolysaccharide-induced microglial activation and neurotoxicity	Zheng et al.	The article investigate applications of phenolic compound in neurodegenerative diseases, not specifically Parkinson's disease
**125**	Epigallocatechin gallate protects nerve growth factor differentiated PC12 cells from oxidative-radical-stress-induced apoptosis through its effect on phosphoinositide 3-kinase/Akt and glycogen synthase kinase-3	Koh et al.	This article is not related to a model of Parkinson’s disease development and doesn’t investigate Parkinson's disease specifically
**124**	Flavonoid-Based Therapies in the Early Management of Neurodegenerative Diseases	Solanki et al.	The article investigate applications of phenolic compound in neurodegenerative diseases, not specifically Parkinson's disease
**124**	Applications of isothermal titration calorimetry in pure and applied researchusurvey of the literature from 2010	Ghai, R.; Falconer, R. J. and Collins, B. M.	The article doesn’t investigate Parkinson's disease and phenolic compounds specifically
**124**	Mechanism of neuroprotective action of the anti-Parkinson	Mandel et al.	The article doesn’t investigate phenolic compounds

	drug rasagiline and its derivatives		
**123**	The art of building multifunctional metal-binding agents from basic molecular scaffolds for the potential application in neurodegenerative diseases	Rodriguez-Rodriguez, C.; Telpoukhovskaia, M. and Orvig, C.	The article doesn’t investigate Parkinson's disease and phenolic compounds specifically
**122**	Targeting dysregulation of brain iron homeostasis in Parkinson's disease by iron chelators	Weinreb et al.	The article doesn’t investigate phenolic compounds
**122**	Natural Antioxidants Protect Neurons in Alzheimer's Disease and Parkinson's Disease	Zhao, B.	The article investigate applications of several antioxidant compounds, not specifically phenolic compounds, in Alzheimer’s and Parkinson’s diseases, not specifically Parkinson's disease
**122**	Cationic albumin conjugated pegylated nanoparticle with its transcytosis ability and little toxicity against blood-brain barrier	Lu et al.	The article doesn’t investigate Parkinson's disease and phenolic compounds specifically
**119**	Cell Signaling Pathways and Iron Chelation in the Neurorestorative Activity of Green Tea Polyphenols: Special Reference to Epigallocatechin Gallate (EGCG)	Mandel et al.	The article investigate applications of phenolic compounds in neurodegenerative diseases, not specifically Parkinson's disease

**117**	The anti-amyloidogenic effect is exerted against Alzheimer's beta-amyloid fibrils in vitro by preferential and reversible binding of flavonoids to the amyloid fibril structure	Hirohata et al.	The article doesn’t investigate Parkinson's disease specifically
**117**	Effect of antioxidant flavanone, naringenin, from Citrus junos on neuroprotection	Heo et al.	This article is not related to a model of Parkinson’s disease development and doesn’t investigate Parkinson's disease specifically
**116**	Rutin inhibits beta-amyloid aggregation and cytotoxicity, attenuates oxidative stress, and decreases the production of nitric oxide and proinflammatory cytokines	Wang et al.	This article is not related to a model of Parkinson’s disease development and doesn’t investigate Parkinson's disease specifically
**116**	Parkinson's disease - Redox mechanisms	Adams, J. D.; Chang, M. L. and Klaidman, L.	The article doesn’t investigate phenolic compounds
**116**	Irreversible inhibition of mitochondrial complex I by 7-(2-aminoethyl)-3,4-dihydro-5-hydroxy-2H-1,4-benzothiazine-3-carboxylic acid (DHBT-1): A putative nigral endotoxin of relevance to Parkinson's disease	Li, H. and Dryhurst, G.	The article doesn’t investigate phenolic compounds
**114**	Ageing and neurodegenerative diseases	Hung et al.	The article investigate the aging factor and doesn’t investigate phenolic compounds. Study neurodegenerative diseases, not specifically Parkinson's disease

**113**	Green tea polyphenols and their potential role in health and disease	Afzal, M.; Safer, A. M. and Menon, M.	The article investigate applications of phenolic compounds in health and diseases, not specifically Parkinson's disease
**110**	Current evidence on the effect of dietary polyphenols intake on chronic diseases	Costa et al.	The article investigate applications of phenolic compounds in chronic diseases, not specifically Parkinson's disease
**110**	The cytotoxic Staphylococcus aureus PSM alpha a3 reveals a cross-alpha amyloid-like fibril	Tayeb-Fligelman et al.	The article doesn’t investigate Parkinson's disease and phenolic compounds specifically
**110**	(-)-Epigallocatechin-3-Gallate (EGCG) Maintains kappa-Casein in Its Pre-Fibrillar State without Redirecting Its Aggregation Pathway	Hudson et al.	This article is not related to a model of Parkinson’s disease development and doesn’t investigate Parkinson's disease specifically
**109**	Expression of NAD(P)H: quinone oxidoreductase in the normal and Parkinsonian substantia nigra	van Muiswinkel et al.	The article doesn’t investigate phenolic compounds
**108**	Dopaminergic neuron-specific oxidative stress caused by dopamine itself	Miyazaki, I. and Asanuma, M.	The article doesn’t investigate phenolic compounds
**107**	Trans-Blood Brain Barrier Delivery of Dopamine-Loaded Nanoparticles Reverses Functional Deficits in Parkinsonian Rats	Pahuja et al.	The article doesn’t investigate phenolic compounds
**107**	Iron-mediated generation of the neurotoxin 6-hydroxydopamine quinone by reaction of fatty acid	Pezzella et al.	The article doesn’t investigate phenolic compounds

	hydroperoxides with dopamine: A possible contributory mechanism for neuronal degeneration in Parkinson's disease		
**105**	CDNA gene expression profile homology of antioxidants and their antiapoptotic and proapoptotic activities in human neuroblastoma cells	Weinreb, O.; Mandel, S. and Youdim, M. B. H.	The article investigate applications of several antioxidant compounds, not specifically phenolic compounds, in antiapoptotic and proapoptotic activities not specifically Parkinson's disease
**104**	Curcuminoids enhance memory in an amyloid-infused rat model of alzheimer's disease	Ahmed, T.; Enam, S. A.; Gilani, A. H.	This article is not related to a model of Parkinson's disease development and doesn't investigate Parkinson's disease specifically
**104**	Challenges Associated with Metal Chelation Therapy in Alzheimer's Disease	Hegde et al.	This article is not related to a model of Parkinson's disease development and doesn't investigate Parkinson's disease specifically
**104**	Screening of various phenolic acids and flavonoid derivatives for their anticholinesterase potential	Orhan et al.	This article is not related to a model of Parkinson's disease development and doesn't investigate Parkinson's disease specifically
**103**	Effects of moderate beer consumption on health and disease: A consensus document	de Gaetano et al.	The article doesn't investigate Parkinson's disease and phenolic compounds specifically
**102**	Potential neuroprotective properties of epigallocatechin-3-gallate (EGCG)	Singh, N. A.; Mandal, A. K. A. and Khan, Z. A.	The article investigate applications of phenolic compound in neuroprotective properties, not specifically Parkinson's disease
**102**	Proteomic identification of dopamine-conjugated proteins from isolated rat brain	Van Laar, Victor S.; Mishizen,	The article doesn't investigate phenolic compounds

	mitochondria and SH-SY5Y cells	Amanda J.; Cascio, Michael; et al	
**101**	Bioactivities of Phenolics by Focusing on Suppression of Chronic Diseases: A Review	Shahidi, F. and Yeo, J. D.	The article investigate applications of phenolic compounds in suppression of chronical diseases, not specifically Parkinson's disease
**101**	Human recombinant monoamine oxidase B as reliable and efficient enzyme source for inhibitor screening	Novaroli et al.	The article doesn’t investigate phenolic compounds and study Alzheimer’s and Parkinson’s diseases, not specifically Parkinson's disease
**100**	Molecular mechanisms of hypolipidemic effects of curcumin	Zingg, J. M.; Hasan, S. T. and Meydani, M.	This article is not related to a model of Parkinson’s disease development and doesn’t investigate Parkinson's disease specifically
**100**	Bromocriptine activates NQO1 via Nrf2-PI3K/Akt signaling: Novel cytoprotective mechanism against oxidative damage	Lim et al.	The article doesn’t investigate phenolic compounds
**100**	Protective effect of protocatechuic acid from Alpinia oxyphylla on hydrogen peroxide-induced oxidative PC12 cell death	Guan et al.	This article is not related to a model of Parkinson’s disease development and doesn’t investigate Parkinson's disease specifically
**100**	Could a loss of alpha-synuclein function put dopaminergic neurons at risk?	Perez, R. G. and Hastings, T. G.	The article doesn’t investigate phenolic compounds
**99**	Recent Advances in the Understanding of the Health Benefits and Molecular Mechanisms Associated with Green Tea Polyphenols	Xing et al.	The article investigate applications of phenolic compounds in health benefits, not specifically Parkinson's disease

**98**	Targeting multiple neurodegenerative diseases etiologies with multimodal-acting green tea catechins	Mandel et al.	The article investigate applications of phenolic compounds in neurodegenerative diseases, not specifically Parkinson's disease
**97**	Alternative Pathways of Human Islet Amyloid Polypeptide Aggregation Distinguished by F-19 Nuclear Magnetic Resonance-Detected Kinetics of Monomer Consumption	Suzuki et al.	This article is not related to a model of Parkinson's disease development and doesn't investigate Parkinson's disease specifically
**97**	Chemistry of periodate-mediated cross-linking of 3,4-dihydroxylphenylalanine-containing molecules to proteins	Liu, B.; Burdine, L. and Kodadek, T.	The article doesn't investigate phenolic compounds
**97**	The cytotoxicity of dopamine may be an artefact of cell culture	Clement et al.	The article doesn't investigate phenolic compounds
**97**	Levodopa toxicity in fetal-rat midbrain neurons in culture - modulation by ascorbic-acid	Mena et al.	The article doesn't investigate phenolic compounds
**95**	The emerging role of nutrition in Parkinson's disease	Seidl et al.	The article investigate applications of several antioxidant compounds, not specifically phenolic compounds
**95**	Differential protective effects of quercetin, resveratrol, rutin and epigallocatechin gallate against mitochondrial	Carrasco-Pozo et al.	This article is not related to a model of Parkinson's disease development and doesn't investigate Parkinson's disease specifically

	dysfunction induced by indomethacin in Caco-2 cells		
**94**	Molecular Mechanisms behind Free Radical Scavengers Function against Oxidative Stress	Ahmadinejad et al.	The article doesn't investigate phenolic compounds. Study oxidative stress in neurodegenerative diseases, not specifically Parkinson's disease
**94**	Autophagy, polyphenols and healthy ageing	Pallauf, K. and Rimbach, G.	The article doesn't investigate Parkinson's disease specifically
**94**	Baicalein Inhibits Formation of alpha-Synuclein Oligomers within Living Cells and Prevents A beta Peptide Fibrillation and Oligomerisation	Lu et al.	The article investigate applications of phenolic compound in Alzheimer's and Parkinson's diseases, not specifically Parkinson's disease
**94**	1-Methyl-4-phenylpyridinium (MPP+)-induced apoptosis and mitochondrial oxidant generation: role of transferrin-receptor-dependent iron and hydrogen peroxide	Kalivendi et al.	The article doesn't investigate phenolic compounds
**94**	Subtype-selective N-methyl-D-aspartate receptor antagonists: Synthesis and biological evaluation of 1-(heteroarylalkynyl)-4-benzylpiperidines	Wright et al.	The article investigate applications of synthetic compounds, not specifically phenolic compounds, in neurodegenerative diseases, not specifically Parkinson's disease
**93**	Mitochondrial membrane permeabilisation by amyloid aggregates and protection by polyphenols	Camilleri et al.	The article investigate applications of phenolic compound in Alzheimer's and Parkinson's diseases, not specifically Parkinson's disease

**93**	Transport of a cancer chemopreventive polyphenol, resveratrol: Interaction with serum albumin and hemoglobin	Lu et al.	The article investigate applications of phenolic compound in cancer chemoprevention, not specifically in Parkinson's disease
**93**	Oxidative and non-oxidative mechanisms of neuronal cell death and apoptosis by L-3,4-dihydroxyphenylalanine (L-DOPA) and dopamine	Pedrosa, R. and Soares-da-Silva, P.	The article doesn't investigate phenolic compounds
**93**	An association between idiopathic Parkinson's disease and polymorphisms of phase II detoxification enzymes: Glutathione S-transferase M1 and quinone oxidoreductase 1 and 2	Harada et al.	The article doesn't investigate phenolic compounds
**92**	Antioxidant activity of 7,8-dihydroxyflavone provides neuroprotection against glutamate-induced toxicity	Chen et al.	The article investigate applications of phenolic compound in neuroprotection, not specifically in Parkinson's disease
**92**	Understanding the Broad-Spectrum Neuroprotective Action Profile of Green Tea Polyphenols in Aging and Neurodegenerative Diseases	Mandel et al.	The article investigate applications of phenolic compounds in neuroprotection, not specifically in Parkinson's disease
**91**	The Role of Catechins in Cellular Responses to Oxidative Stress	Bernatoniene, J. and Kopustinskiene, D. M.	The article investigate applications of phenolic compound in oxidative stress, not specifically in Parkinson's disease

**91**	Analysis of the Inhibition and Remodeling of Islet Amyloid Polypeptide Amyloid Fibers by Flavanols	Cao, P. and Raleigh, D. P.	This article is not related to a model of Parkinson's disease development and doesn't investigate Parkinson's disease specifically
**91**	Potential role of green tea catechins in various disease therapies: Progress and promise	Mak, J. C. W.	The article investigate applications of phenolic compounds in disease therapies, not specifically Parkinson's disease
**91**	Protective effect of sulforaphane against dopaminergic cell death	Han et al.	The article doesn't investigate phenolic compounds. Study dopaminergic cell death, not specifically in Parkinson's disease
**90**	Biomedical effects of grape products	Vislocky, L. M. and Fernandez, M. L.	The article investigate applications of phenolic compounds in biomedical effects, not specifically in Parkinson's disease
**90**	Unique properties of polyphenol stilbenes in the brain: More than direct antioxidant actions; Gene/protein regulatory activity	Dore, S.	The article investigate applications of phenolic compounds in biomedical effects, not specifically in Parkinson's disease
**90**	Mitochondrial DNA mutations in complex I and tRNA genes in Parkinson's disease	Simon et al.	The article doesn't investigate phenolic compounds.
**89**	Resveratrol Improves Motoneuron Function and Extends Survival in SOD1(G93A) ALS Mice	Mancuso et al.	This article is not related to a model of Parkinson's disease development and doesn't investigate Parkinson's disease specifically
**89**	Coenzyme Q10 in Neuromuscular and Neurodegenerative Disorders	Mancuso et al.	The article doesn't investigate phenolic compounds, only Coenzyme Q10, not specifically in Parkinson's disease

**89**	The transcription factor Nrf2 as a new therapeutic target in Parkinson's disease	Cuadrado, A; Moreno-Murciano, P. and Pedraza-Chaverri, J.	The article doesn’t investigate phenolic compounds
**89**	Role of oxidative changes in the degeneration of dopamine terminals after injection of neurotoxic levels of dopamine	Rabinovic, A. D.; Lewis, D. A. and Hastings, T. G.	The article doesn’t investigate phenolic compounds
**88**	Healthy Effects of Plant Polyphenols: Molecular Mechanisms	Leri et al.	The article investigate applications of phenolic compounds in healthy effects, not specifically in Parkinson's disease
**88**	Antioxidant protection: A promising therapeutic intervention in neurodegenerative disease	Ghosh, N.; Ghosh, R. and Mandal, S. C.	The article investigate applications of antioxidant compounds, not specifically phenolic compounds, in neurodegenerative diseases, not specifically Parkinson's disease
**88**	The effect of epigallocatechin gallate on suppressing disease progression of ALS model mice	Koh et al.	This article is not related to a model of Parkinson’s disease development and doesn’t investigate Parkinson's disease specifically
**87**	The Polyphenol EGCG Inhibits Amyloid Formation Less Efficiently at Phospholipid Interfaces than in Bulk Solution	Engel et al.	The article investigate applications of phenolic compound in inhibition of amyloid formation, not specifically in Parkinson's disease
**87**	Neurorescue activity, APP regulation and amyloid-beta peptide reduction by novel multi-functional brain permeable iron- chelating-	Avramovich-Tirosh et al.	This article is not related to a model of Parkinson’s disease development and doesn’t investigate Parkinson's disease specifically

	antioxidants, m-30 and green tea polyphenol, EGCG		
**87**	Dieldrin exposure induces oxidative damage in the mouse nigrostriatal dopamine system	Hatcher et al.	The article doesn’t investigate phenolic compounds.
**87**	Drug treatment of Parkinson's disease - Time for phase II	Drukarch, B. and van Muiswinkel, F. L.	The article investigate applications of drug compounds, not specifically phenolic compounds
**87**	Inhibition of glutamate transport in synaptosomes by dopamine oxidation and reactive oxygen species	Berman, S. B. and Hastings, T. G.	The article doesn’t investigate phenolic compounds. Study inhibition of glutamate transport in synaptosomes, not specifically in Parkinson's disease
**87**	Further insights into the influence of L-cysteine on the oxidation chemistry of dopamine: Reaction pathways of potential relevance to Parkinson's disease	Shen, X. M. and Dryhurst, G.	The article doesn’t investigate phenolic compounds
**86**	Curcumin and neurodegenerative diseases: a perspective	Darvesh et al	The article investigate applications of phenolic compound in neurodegenerative diseases, not specifically in Parkinson's disease
**86**	Baicalein protects HT22 murine hippocampal neuronal cells against endoplasmic reticulum stress-induced apoptosis through inhibition of reactive oxygen species production and CHOP induction	Choi et al.	The article investigate applications of phenolic compound in neuroprotective effects, not specifically in Parkinson's disease

**86**	Inhibition of alpha-synuclein fibrillization by dopamine analogs via reaction with the amino groups of alpha-synuclein	Li et al.	The article investigate applications of synthetic compounds, not specifically phenolic compounds
**85**	Levodopa induces apoptosis in cultured neuronal cells - A possible accelerator of nigrostriatal degeneration in Parkinson's disease?	Ziv et al.	The article doesn’t investigate phenolic compounds
**84**	Resveratrol: A Focus on Several Neurodegenerative Diseases	Tellone et al.	The article investigate applications of phenolic compound in neurodegenerative diseases, not specifically in Parkinson's disease
**84**	Characterization of the antioxidant functions of flavonoids and proanthocyanidins in Mauritian black teas	Luximon-Ramma et al.	The experiments tested in the article are nonspecific to indicate outcomes related only to Parkinson's disease
**84**	Mitochondria Play a Central Role in Estrogen-Induced Neuroprotection	Simpkins et al.	The article doesn’t investigate phenolic compounds. Study estrogen-induced neuroprotection in mitochondria, not specifically in Parkinson's disease
**84**	Glia protect fetal midbrain dopamine neurons in culture from L-DOPA toxicity through multiple mechanisms	Mena et al.	The article doesn’t investigate phenolic compounds
**84**	Glia conditioned medium protects fetal rat midbrain neurones in culture from L-DOPA toxicity	Mena et al.	The article doesn’t investigate phenolic compounds

**83**	The reaction of alpha-synuclein with tyrosinase - Possible implications for Parkinson disease	Tessari et al.	The article doesn't investigate phenolic compounds
**83**	Initiation of cancer and other diseases by catechol ortho-quinones: a unifying mechanism	Cavalieri, E. L.; Rogan, E. G. and Chakravarti, D.	The article doesn't investigate Parkinson's disease and phenolic compounds specifically
**82**	Bioactivity of Olive Oil Phenols in Neuroprotection	Angeloni et al.	The article doesn't investigate Parkinson's disease specifically
**82**	Neuroprotective Effects of Zonisamide Target Astrocyte	Asanuma et al.	The article doesn't investigate phenolic compounds
**82**	Monoamine Oxidase Inhibitors as Neuroprotective Agents in Age-Dependent Neurodegenerative Disorders	Naoi, M. and Maruyama, W.	The article investigate applications of neuroprotective compounds, not specifically phenolic compounds, in neurodegenerative disorders, not specifically in Parkinson's disease
**82**	Fruit polyphenols and their effects on neuronal signaling and behavior in senescence	Joseph, J. A.; Shukitt-Hale, B. and Lau, F. C.	The article investigate applications of phenolic compound in neuro health, not specifically in Parkinson's disease
**81**	Morin hydrate inhibits amyloid formation by islet amyloid polypeptide and disaggregates amyloid fibers	Noor, H.; Cao, P. and Raleigh, D. P.	This article is not related to a model of Parkinson's disease development and doesn't investigate Parkinson's disease specifically
**81**	Unifying mechanism in the initiation of cancer and other diseases by catechol quinones	Cavalieri, E. L. and Rogan, E. G.	The article investigate applications of neuroprotective compounds, not specifically phenolic compounds, in neurodegenerative disorders, not specifically in Parkinson's disease
**80**	Grape Derived Polyphenols Attenuate Tau Neuropathology	Wang et al.	This article is not related to a model of Parkinson's disease development and doesn't investigate Parkinson's disease specifically

	in a Mouse Model of Alzheimer's Disease		
**80**	Protection by the NDI1 Gene against Neurodegeneration in a Rotenone Rat Model of Parkinson's Disease	Marella et al.	The article doesn’t investigate phenolic compounds.
**80**	Tyrosinase exacerbates dopamine toxicity but is not genetically associated with Parkinson's disease	Greggio et al	The article doesn’t investigate phenolic compounds
**79**	The genus Anemarrhena Bunge: A review on ethnopharmacology, phytochemistry and pharmacology	Wang et al.	The article investigate applications of neuroprotective compounds, not specifically phenolic compounds, in neurodegenerative disorders, not specifically in Parkinson's disease
**79**	Selective MAO-B inhibitors: a lesson from natural products	Carradori et al.	The article investigate applications of natural products, not specifically phenolic compounds, in neurodegenerative disorders, not specifically in Parkinson's disease
**79**	Oxidation Chemistry of Catecholamines and Neuronal Degeneration: An Update	Napolitano, A.; Manini, P. and d'Ischia, M.	The article doesn’t investigate Parkinson's disease
**79**	A potential role for cyclized quinones derived from dopamine, DOPA, and 3,4-dihydroxyphenylacetic acid in proteasomal inhibition	Zafar, K. S.; Siegel, D. and Ross, D.	The article doesn’t investigate Parkinson's disease
**79**	p-quinone mediates 6-hydroxydopamine-induced dopaminergic neuronal death	Izumi et al.	The article doesn’t investigate phenolic compounds

	and ferrous iron accelerates the conversion of p-quinone into melanin extracellularly		
**78**	Medicinal uses, phytochemistry and pharmacology of the genus Uncaria	Zhang et al.	The article doesn’t investigate Parkinson's disease and phenolic compounds specifically
**78**	Food, Nutrigenomics, and Neurodegeneration-Neuroprotection by What You Eat!	Virmani et al.	The article investigate applications of natural products, not specifically phenolic compounds, in neuroprotection, not specifically in Parkinson's disease
**78**	Obligatory role for complex I inhibition in the dopaminergic neurotoxicity of 1-methyl-4-phenyl-1,2,3,6-tetrahydropyridine (MPTP)	Richardson et al.	The article doesn’t investigate phenolic compounds
**78**	Neuroprotection by bioactive components in medicinal and food plant extracts	Aruoma, O. I.; Bahorun, T. and Jen, L. S.	The article investigate applications of bioactive compounds, not specifically phenolic compounds, in neuroprotection, not specifically in Parkinson's disease
**77**	Antioxidants: Scientific Literature Landscape Analysis	Yeung et al.	The article investigate applications of antioxidant compounds, not specifically phenolic compounds, in neuroprotection, not specifically in Parkinson's disease
**77**	Cellular and molecular mechanisms of antioxidants in Parkinson's disease	Jairo et al.	The article investigate applications of antioxidant compounds, not specifically phenolic compounds, in Parkinson's disease
**77**	A beta(1-42) Aggregates into Non-Toxic Amyloid Assemblies in the Presence of	Rigacci et al.	This article is not related to a model of Parkinson’s disease development and doesn’t investigate Parkinson's disease specifically

	the Natural Polyphenol Oleuropein Aglycon		
**77**	Ligand binding to distinct states diverts aggregation of an amyloid-forming protein	Woods et al.	This article is not related to a model of Parkinson’s disease development and doesn’t investigate phenolic compounds
**77**	Icariin attenuates beta-amyloid-induced neurotoxicity by inhibition of tau protein hyperphosphorylation in PC12 cells	Zeng et al.	This article is not related to a model of Parkinson’s disease development and doesn’t investigate Parkinson's disease specifically
**77**	Genistein, a natural phytoestrogen from soy, relieves neuropathic pain following chronic constriction sciatic nerve injury in mice: anti-inflammatory and antioxidant activity	Valsecchi et al.	This article is not related to a model of Parkinson’s disease development and doesn’t investigate Parkinson's disease specifically
**77**	Proteomic analysis of rat brain mitochondria following exposure to dopamine quinone: Implications for Parkinson disease	Van Laar et al.	The article doesn’t investigate phenolic compounds
**77**	Catechol quinones of estrogens in the initiation of breast, prostate, and other human cancers - Keynote lecture	Cavalieri, E. and Rogan, E.	This article is not related to a model of Parkinson’s disease development and doesn’t investigate phenolic compounds
**76**	Mitochondrial dysfunction and cell death in neurodegenerative diseases through nitroxidative stress	Akbar et al.	The article doesn’t investigate Parkinson's disease and phenolic compounds specifically

**76**	Structural insight into the type-II mitochondrial NADH dehydrogenases	Feng et al.	The article doesn’t investigate phenolic compounds
**76**	Neuroprotective Effects of Chronic Hesperetin Administration in Mice	Choi, E. J. and Ahn, W. S.	The article investigate applications of phenolic compound in neuroprotection, not specifically in Parkinson's disease
**76**	An electrochemical approach for detecting copper-chelating properties of flavonoids using disposable pencil graphite electrodes: Possible implications in copper-mediated illnesses	Vestergaard, M.; Kerman, K. and Tamiya, E.	The article investigate applications of phenolic compounds in neuroprotection, not specifically in Parkinson's disease
**75**	Hesperetin, a Citrus Flavonoid, Attenuates LPS-Induced Neuroinflammation, Apoptosis and Memory Impairments by Modulating TLR4/NF-kappa B Signaling	Muhammad et al.	This article is not related to a model of Parkinson’s disease development and doesn’t investigate Parkinson's disease specifically
**75**	Novel tactics for neuroprotection in Parkinson's disease: Role of antibiotics, polyphenols and neuropeptides	Reglodi et al.	The article investigate applications of compounds in neuroprotection, not specifically phenolic compounds for neuroprotection in Parkinson's disease
**75**	Olive polyphenols: new promising agents to combat aging-associated neurodegeneration	Casamenti, F. and Stefani, M.	The article investigate applications of phenolic compound in neurodegeneration, not specifically in Parkinson's disease
**75**	Structural Insights into Ubiquinone Biosynthesis in Membranes	Cheng, W. and Li, W.	This article is not related to a model of Parkinson’s disease development and doesn’t investigate phenolic compounds

**75**	Structural and mechanistic basis behind the inhibitory interaction of PcTS on alpha-synuclein amyloid fibril formation	Lamberto et al.	The article doesn’t investigate phenolic compounds
**75**	Protein Reactivity of 3,4-Dihydroxyphenylacetaldehyde, a Toxic Dopamine Metabolite, Is Dependent on Both the Aldehyde and the Catechol	Rees et al.	The article doesn’t investigate phenolic compounds
**75**	Enhanced nad(p)h-quinone reductase-activity prevents glutamate toxicity produced by oxidative stress	Murphy, T. H.; Delong, M. J. and Coyle, J. T.	The article doesn’t investigate phenolic compounds
**74**	Flavonoids as acetylcholinesterase inhibitors: Current therapeutic standing and future prospects	Khan et al.	The article investigate applications of phenolic compound in acetylcholinesterase inhibition, not specifically in Parkinson's disease
**74**	Role of the Keap1/Nrf2 pathway in neurodegenerative diseases	Yamazaki et al.	The article doesn’t investigate phenolic compounds
**74**	Gallic acid, one of the components in many plant tissues, is a potential inhibitor for insulin amyloid fibril formation	Jayamani, J. and Shanmugam, G.	This article is not related to a model of Parkinson’s disease development and doesn’t investigate Parkinson's disease specifically
**74**	Mitochondrial dysfunction mediated by quinone oxidation products of dopamine: Implications in dopamine	Jana et al.	The article doesn’t investigate phenolic compounds

	cytotoxicity and pathogenesis of Parkinson's disease		
**74**	The Crystal Structure of the Green Tea Polyphenol (-)-Epigallocatechin Gallate-Transthyretin Complex Reveals a Novel Binding Site Distinct from the Thyroxine Binding Site	Miyata et al.	This article is not related to a model of Parkinson’s disease development and doesn’t investigate Parkinson's disease
**74**	Tyrosinase-Expressing Neuronal Cell Line as in Vitro Model of Parkinson's Disease	Hasegawa, T.	The article doesn’t investigate phenolic compounds
**74**	Sulforaphane as an inducer of glutathione prevents oxidative stress-induced cell death in a dopaminergic-like neuroblastoma cell line	Tarozzi et al.	The article doesn’t investigate phenolic compounds
**74**	Dopamine-derived dopaminochrome promotes H2O2 release at mitochondrial complex I - Stimulation by rotenone, control by Ca2+, and relevance to Parkinson disease	Zoccarato, F.; Toscano, P. and Alexandre, A.	The article doesn’t investigate phenolic compounds
**73**	Anti-Aging Implications of Astragalus Membranaceus (Huangqi): A Well-Known Chinese Tonic	Liu, P.; Zhao, H. and Luo, Y.	The article investigate applications of compounds, not specifically phenolic compounds for anti-aging effects, not in Parkinson's disease specifically
**73**	Hydrogen sulfide in pharmacology and medicine - An update	Beltowski, J.	The article investigate applications of compounds, not specifically phenolic

			compounds for neuroprotection, not in Parkinson's disease specifically
**73**	Protective effects of hyperoside (quercetin-3-o-galactoside) to PC12 cells against cytotoxicity induced by hydrogen peroxide and tert-butyl hydroperoxide	Liu et al.	The article investigate applications of phenolic compounds for neuroprotection, not in Parkinson's disease specifically
**73**	The neurotoxicity of glutamate, dopamine, iron and reactive oxygen species: Functional interrelationships in health and disease: A review - discussion	Smythies, J.	The article investigate applications of compounds, not specifically phenolic compounds, for neuroprotection, not in Parkinson's disease specifically
**73**	Iron- and manganese-catalyzed autoxidation of dopamine in the presence of L-cysteine: Possible insights into iron- and manganese-mediated dopaminergic neurotoxicity	Shen, X. M. and Dryhurst, G.	The article doesn’t investigate phenolic compounds
**72**	Annona muricata: A comprehensive review on its traditional medicinal uses, phytochemicals, pharmacological activities, mechanisms of action and toxicity	Coria-Tellez et al.	The article investigate applications of bioactive compounds, not specifically phenolic compounds for neuroprotection, not in Parkinson's disease specifically
**72**	Sensorial properties of red wine polyphenols: Astringency and bitterness	Soares et al.	The article investigate applications of phenolic compounds in neuroprotection, not for Parkinson's disease specifically

**72**	Anti-Parkinsonian drug discovery from herbal medicines: What have we got from neurotoxic models?	Song et al.	The article investigate applications of herbal compounds, not specifically phenolic compounds in Parkinson's disease
**72**	Wine Polyphenols: Potential Agents in Neuroprotection	Basli et al.	The article investigate applications of vine phenolic compounds in neuroprotection, not specifically for Parkinson's disease
**72**	The Yeast Complex I Equivalent NADH Dehydrogenase Rescues pink1 Mutants	Vilain et al.	The article doesn’t investigate phenolic compounds
**71**	The Neuroprotective Effects of Phenolic Acids: Molecular Mechanism of Action	Szwajgier, D.; Borowiec, K. and Pustelniak, K.	The article investigate applications of phenolic compounds in neuroprotection, not specifically for Parkinson's disease
**71**	L-cysteine capped ZnS:Mn quantum dots for room-temperature detection of dopamine with high sensitivity and selectivity	Diaz-Diestra et al.	The article doesn’t investigate phenolic compounds
**71**	Flavonoid transport across RBE4 cells: A blood-brain barrier model	Faria et al.	The article investigate applications of phenolic compounds in neuroprotection, not specifically for Parkinson's disease
**71**	Dopamine-dependent cytotoxicity of tetrahydrobiopterin: a possible mechanism for selective neurodegeneration in Parkinson's disease	Choi et al.	The article doesn’t investigate phenolic compounds
**71**	Enzyme activity of macrophage migration	Matsunaga et al.	The article investigate applications of compounds, not specifically phenolic

	inhibitory factor toward oxidized catecholamines		compounds in neuroprotection, not for Parkinson's disease specifically
**70**	Natural product-based amyloid inhibitors	Velander et al.	The article investigate applications of natural products, not specifically phenolic compounds for amyloid inhibition, not in Parkinson's disease specifically
**70**	Effects of several quinones on insulin aggregation	Gong et al.	This article is not related to a model of Parkinson’s disease development and doesn’t investigate phenolic compounds
**70**	Target-based selection of flavonoids for neurodegenerative disorders	Jones et al.	The article investigate applications of phenolic compounds in neurodegenerative disorders, not specifically for Parkinson's disease
**69**	Rationally Designed Multi-Targeted Agents Against Neurodegenerative Diseases	Geldenhuys, W. J. andVan der Schyf, C. J.	This article is not related to a model of Parkinson’s disease development and doesn’t investigate phenolic compounds
**69**	Effect of estrogen receptor agonists treatment in MPTP mice: evidence of neuroprotection by an ER alpha agonist	D'Astous, M.; Morissette, M. and Di Paolo, T.	The article doesn’t investigate phenolic compounds
**69**	Effects of UV treatment on the proline-linked pentose phosphate pathway for phenolics and L-DOPA synthesis in dark germinated Vicia faba	Shetty, P.; Atallah, M. T. and Shetty, K.	The article investigate applications of synthetic compounds, not specifically phenolic compounds
**68**	Sulforaphane protects against rotenone-induced neurotoxicity in vivo: Involvement of the	Zhou et al.	The article doesn’t investigate phenolic compounds

	mTOR, Nrf2, and autophagy pathways		
**68**	Umbelliferone ameliorates cerebral ischemia-reperfusion injury via upregulating the PPAR gamma expression and suppressing TXNIP/NLRP3 inflammasome	Wang et al.	This article is not related to a model of Parkinson’s disease development
**68**	Gallic acid is the major component of grape seed extract that inhibits amyloid fibril formation	Liu et al.	This article is not related to a model of Parkinson’s disease development
**68**	Role of endogenous glutathione in the oxidation of dopamine	Rabinovic, A. D. and Hastings, T. G.	The article doesn’t investigate phenolic compounds
**67**	Neuroprotective effects of berry fruits on neurodegenerative diseases	Subash et al.	The article investigate applications of berry fruits, not specifically phenolic compounds in neurodegenerative diseases, not for Parkinson's disease specifically
**67**	Site Specific Interaction of the Polyphenol EGCG with the SEVI Amyloid Precursor Peptide PAP(248-286)	Popovych et al.	This article is not related to a model of Parkinson’s disease development
**67**	In vivo complementation of complex I by the yeast Ndi1 enzyme - Possible application for treatment of Parkinson disease	Seo et al.	The article doesn’t investigate phenolic compounds
**67**	Inhibition of brain mitochondrial respiration by	Gluck, M. R. and Zeevalk, G. D.	The article doesn’t investigate phenolic compounds

	dopamine and its metabolites: implications for Parkinson's disease and catecholamine-associated diseases		
**67**	Modifying effects of dietary capsaicin and rotenone on 4-nitroquinoline 1-oxide-induced rat tongue carcinogenesis	Tanaka et al.	This article is not related to a model of Parkinson’s disease development
**66**	Neuroprotective Effect of Fisetin Against Amyloid-Beta-Induced Cognitive/Synaptic Dysfunction, Neuroinflammation, and Neurodegeneration in Adult Mice	Ahmad et al.	This article is not related to a model of Parkinson’s disease development
**66**	Chronic consumption of flavanone-rich orange juice is associated with cognitive benefits: an 8-wk, randomized, double-blind, placebo-controlled trial in healthy older adults	Kean et al.	This article is not related to a model of Parkinson’s disease development
**66**	Effect of natural exogenous antioxidants on aging and on neurodegenerative diseases	Guerra-Araiza et al.	The article investigate applications of antioxidant compounds, not specifically phenolic compounds in neurodegenerative diseases, not for Parkinson's disease specifically
**66**	SOD1 and DJ-1 Converge at Nrf2 Pathway: A Clue for Antioxidant Therapeutic Potential in Neurodegeneration	Milani et al.	The article investigate applications of phenolic compounds in Parkinson’s and sclerosis diseases, not for Parkinson's disease specifically

**66**	Epigallocatechin-3-Gallate as a Potential Therapeutic Drug for TTR-Related Amyloidosis: "In Vivo" Evidence from FAP Mice Models	Ferreira, N.; Saraiva, M. J.; Almeida, M. R.	This article is not related to a model of Parkinson’s disease development
**66**	Pleiotropic Protective Effects of Phytochemicals in Alzheimer's Disease	Davinelli et al.	This article is not related to a model of Parkinson’s disease development
**66**	Berry anthocyanins and their aglycons inhibit monoamine oxidases A and B	Dreiseitel et al.	The article investigate applications of phenolic compounds in neurodegenerative diseases, not for Parkinson's disease specifically
**65**	N-Palmitoylethanolamine and Neuroinflammation: a Novel Therapeutic Strategy of Resolution	Skaper et al.	The article investigate applications of compounds, not specifically phenolic compounds in neuroinflamation, not for Parkinson's disease specifically
**65**	The Parkinson's-associated protein DJ-1 regulates the 20S proteasome	Moscovitz et al.	The article doesn’t investigate phenolic compounds
**65**	Apigenin protects HT22 murine hippocampal neuronal cells against endoplasmic reticulum stress-induced apoptosis	Choi et al.	This article is not related to a model of Parkinson’s disease development
**65**	The structure of dopamine induced alpha-synuclein oligomers	Rekas et al.	The article doesn’t investigate phenolic compounds
**65**	The Flavonoid Glycosides, Myricitrin, Gossypin and Naringin Exert Anxiolytic Action in Mice	Fernandez et al.	This article is not related to a model of Parkinson’s disease development

**64**	Lifestyle, health and disease prevention: the underlying mechanisms	Weisburger, J. H.	The article investigate applications of compounds, not specifically phenolic compounds in disease prevention, not for Parkinson's disease specifically
**63**	Are Astrocytes the Predominant Cell Type for Activation of Nrf2 in Aging and Neurodegeneration?	Liddell, J. R.	The article doesn't investigate phenolic compounds
**63**	Revisiting an ancient spice with medicinal purposes: Cinnamon	Ribeiro-Santos et al.	The article investigate applications of compounds, not specifically phenolic compounds in medicinal purposes, not for Parkinson's disease specifically
**63**	Safety, tolerability, and pharmacokinetics of a single ascending dose of baicalein chewable tablets in healthy subjects	Li et al.	The article doesn't investigate Parkinson's disease
**63**	Oxidation of 3,4-Dihydroxyphenylacetaldehyde, a Toxic Dopaminergic Metabolite, to a Semiquinone Radical and an ortho-Quinone	Anderson et al.	The article doesn't investigate phenolic compounds
**63**	Dopamine quinones interact with a-synuclein to form unstructured adducts	Bisaglia et al.	The article doesn't investigate phenolic compounds
**63**	Nrf2 Activators Provide Neuroprotection Against 6-Hydroxydopamine Toxicity in Rat Organotypic Nigrostriatal Cocultures	Siebert et al.	The article doesn't investigate phenolic compounds

**63**	Gene and protein expression profiles of anti- and pro-apoptotic actions of dopamine, R-apomorphine, green tea polyphenol (-)-epigallocatechine-3-gallate, and melatonin	Weinreb, O.; Mandel, S. and Youdim, M. B. H.	The article investigate applications of compounds, not specifically phenolic compounds
**62**	Use of Curcumin, a Natural Polyphenol for Targeting Molecular Pathways in Treating Age-Related Neurodegenerative Diseases	Maiti, P. and Dunbar, G. L.	The article investigate applications of phenolic compounds in neurodegenerative diseases, not for Parkinson's disease specifically
**62**	Synergistic interactions among flavonoids and acetogenins in Graviola (Annona muricata) leaves confer protection against prostate cancer	Yang et al.	This article is not related to a model of Parkinson's disease development
**62**	Flavones from Root of Scutellaria Baicalensis Georgi: Drugs of the Future in Neurodegeneration?	Gasiorowski et al.	The article investigate applications of phenolic compounds in neurodegenerative diseases, not for Parkinson's disease specifically
**62**	Vesicular monoamine transporter 2 regulates the sensitivity of rat dopaminergic neurons to disturbed cytosolic dopamine levels	Vergo et al.	The article doesn't investigate phenolic compounds
**61**	Rutin as a Potent Antioxidant: Implications for Neurodegenerative Disorders	Enogieru et al.	The article investigate applications of phenolic compounds in neurodegenerative diseases, not for Parkinson's disease specifically

**61**	Polyphenols as Therapeutic Molecules in Alzheimer's Disease Through Modulating Amyloid Pathways	Lakey-Beitia et al.	This article is not related to a model of Parkinson’s disease development
**61**	Natural polyphenols binding to amyloid: A broad class of compounds to treat different human amyloid diseases	Ngoungoure et al.	The article investigate applications of phenolic compounds in amyloid diseases, not for Parkinson's disease specifically
**61**	Mechanism of cell death caused by complex I defects in a rat dopaminergic cell line	Marella et al.	The article doesn’t investigate phenolic compounds
**61**	Pesticide exposure on southwestern Taiwanese with MnSOD and NQO1 polymorphisms is associated with increased risk of Parkinson's disease	Fong et al.	The article doesn’t investigate phenolic compounds
**61**	Catechol oxidation by peroxidase-positive astrocytes in primary culture - an electron-spin-resonance study	Schipper, H. M.; Kotake, Y. and Janzen, E. G.	The article doesn’t investigate phenolic compounds. Investigate applications in catechol oxidation, not for Parkinson's disease specifically
**60**	Quercetin attenuates neuronal death against aluminum-induced neurodegeneration in the rat hippocampus	Sharma et al.	The article investigate applications of phenolic compound in neurodegeneration, not for Parkinson's disease specifically
**60**	A Review of the Cognitive Effects Observed in Humans Following Acute Supplementation with Flavonoids, and Their	Bell et al.	The article investigate applications of phenolic compounds in cognitive effects, not for Parkinson's disease specifically

	Associated Mechanisms of Action		
**60**	Immobilization stress causes increases in tetrahydrobiopterin, dopamine, and neuromelanin and oxidative damage in the nigrostriatal system	Kim et al.	The article doesn’t investigate phenolic compounds
**60**	Inhibition of rat brain mitochondrial electron transport chain activity by dopamine oxidation products during extended in vitro incubation: Implications for Parkinson's disease	Khan et al.	The article doesn’t investigate phenolic compounds
**60**	Coenzyme Q cytoprotective mechanisms for mitochondrial complex I cytopathies involves NAD(P)H: Quinone oxidoreductase 1(NQO1)	Chan et al.	The article doesn’t investigate phenolic compounds
**60**	Synthesis, redox properties, in vivo formation, and neurobehavioral effects of N-acetylcysteinyl conjugates of dopamine: Possible metabolites of relevance to Parkinson's disease	Shen et al.	The article doesn’t investigate phenolic compounds
**59**	Curcumin Pyrazole and its derivative (N-(3-Nitrophenylpyrazole)	Ahsan et al.	The article investigate applications of synthetic compounds, not specifically phenolic compounds

	Curcumin inhibit aggregation, disrupt fibrils and modulate toxicity of Wild type and Mutant alpha-Synuclein		
**59**	Targeting multiple pathogenic mechanisms with polyphenols for the treatment of Alzheimer's disease-experimental approach and therapeutic implications	Wang et al.	This article is not related to a model of Parkinson's disease development
**59**	Astrocyte-Derived Metallothionein Protects Dopaminergic Neurons from Dopamine Quinone Toxicity	Miyazaki et al.	The article doesn't investigate phenolic compounds
**59**	Common anti-apoptotic roles of parkin and alpha-synuclein in human dopaminergic cells	Machida et al.	The article doesn't investigate phenolic compounds
**59**	Green tea polyphenol (-)-epigallocatechin-3-gallate protects rat PC12 cells from apoptosis induced by serum withdrawal	Mandel et al.	The article investigate applications of phenolic compounds in apoptosis, not for Parkinson's disease specifically
**59**	Levodopa-induced neurotoxicity - Does it represent a problem for the treatment of Parkinson's disease?	Fahn, S.	The article doesn't investigate phenolic compounds
**59**	Health functionality of apigenin: A review	Ali et al.	The article investigate applications of phenolic compound in health functionality, not for Parkinson's disease specifically

**58**	Focusing on New Monoamine Oxidase Inhibitors: Differently Substituted Coumarins As An Interesting Scaffold	Matos et al.	The article investigate applications of synthetic compounds, not specifically phenolic compounds, in neurodegenerative diseases, not specifically Parkinson's disease
**58**	Neuroprotective Mechanism of Mitochondrial Ferritin on 6-Hydroxydopamine-Induced Dopaminergic Cell Damage: Implication for Neuroprotection in Parkinson's Disease	Shi et al.	The article doesn’t investigate phenolic compounds
**58**	Cellular oligomerization of alpha-synuclein is determined by the interaction of oxidized catechols with a c-terminal sequence	Mazzulli et al.	The article doesn’t investigate phenolic compounds
**58**	On the function of neuromelanin	Smythies, J.	The article doesn’t investigate phenolic compounds
**57**	Regulation of Toll-Like Receptor (TLR) Signaling Pathway by Polyphenols in the Treatment of Age-Linked Neurodegenerative Diseases: Focus on TLR4 Signaling	Azam et al.	The article investigate applications of phenolic compound in neurodegenerative diseases, not for Parkinson's disease specifically
**57**	The Potential Use of Plant Natural Products and Plant Extracts with Antioxidant Properties for the Prevention/Treatment of Neurodegenerative Diseases:	Pohl, F. and Lin, P. K. T.	The article investigate applications of natural products and plant extracts, not specifically phenolic compounds, in neurodegenerative diseases, not for Parkinson's disease specifically

	In Vitro, In Vivo and Clinical Trials		
**57**	Neurodegenerative diseases: From available treatments to prospective herbal therapy	Solanki, I.; Parihar, P. and Parihar, M. S.	The article investigate applications of phenolic compounds in treatments to prospective herbal therapy, not for Parkinson's disease specifically
**57**	Implication of coumarins towards central nervous system disorders	Skalicka-Wozniak et al.	The article investigate applications of phenolic compounds in central nervous system disorders, not for Parkinson's disease specifically
**57**	Naturally occurring polyphenolic inhibitors of amyloid beta aggregation	Churches et al.	This article is not related to a model of Parkinson’s disease development
**57**	Effects of anthocyanins on psychological stress-induced oxidative stress and neurotransmitter status	Rahman et al.	This article is not related to a model of Parkinson’s disease development
**57**	Concurrent administration of Neu2000 and lithium produces marked improvement of motor neuron survival, motor function, and mortality in a mouse model of amyotrophic lateral sclerosis	Shin et al.	This article is not related to a model of Parkinson’s disease development
**57**	Quinone formation as dopaminergic neuron-specific oxidative stress in the pathogenesis of sporadic Parkinson's disease and neurotoxin-induced parkinsonism	Asanuma et al.	The article doesn’t investigate phenolic compound

**56**	Polyphenol Health Effects on Cardiovascular and Neurodegenerative Disorders: A Review and Meta-Analysis	Poti et al.	The article investigate applications of phenolic compounds in neurodegenerative disorders, not for Parkinson's disease specifically
**56**	Efficient Inhibition of Protein Aggregation, Disintegration of Aggregates, and Lowering of Cytotoxicity by Green Tea Polyphenol-Based Self-Assembled Polymer Nanoparticles	Debnath et al.	This article is not related to a model of Parkinson’s disease development
**56**	Parkinsonian toxin-induced oxidative stress inhibits basal autophagy in astrocytes via NQO2/quinone oxidoreductase 2: Implications for neuroprotection	Janda et al.	The article doesn’t investigate phenolic compounds
**55**	Therapeutic role of sirtuins in neurodegenerative disease and their modulation by polyphenols	Ajami et al.	The article doesn’t investigate phenolic compounds and study neurodegenerative disease, not Parkinson's disease specifically
**55**	Ayurvedic medicinal plants for Alzheimer's disease: a review	Rao et al.	This article is not related to a model of Parkinson’s disease development
**55**	Luteolin inhibits cytokine expression in endotoxin/cytokine-stimulated microglia	Kao et al.	The article investigate applications of phenolic compound in neurodegenerative disorders, not for Parkinson's disease specifically
**55**	Tea Catechins Induce the Conversion of Preformed	He et al.	The article investigate applications of phenolic compounds in neurodegenerative disorders, not for Parkinson's disease specifically

	Lysozyme Amyloid Fibrils to Amorphous Aggregates		
**55**	Changes in endoplasmic reticulum stress proteins and aldolase A in cells exposed to dopamine	Dukes et al.	The article doesn’t investigate phenolic compounds
**55**	Redox mechanisms at the glutamate synapse and their significance: a review	Smythies, J.	The article investigate applications of compounds, not specifically phenolic compounds, in neurodegenerative disorders, not for Parkinson's disease specifically
**55**	Levodopa toxicity and apoptosis	Melamed et al.	The article doesn’t investigate phenolic compounds
**55**	Mechanism of the manganese-catalyzed autoxidation of dopamine	Lloyd, R. V.	The article doesn’t investigate phenolic compounds
**54**	Flavonoids as Therapeutic Agents in Alzheimer's and Parkinson's Diseases: A Systematic Review of Preclinical Evidences	de Andrade et al.	The article investigate applications of phenolic compound in neurodegenerative disorders, not for Parkinson's disease specifically
**54**	Oxidative stress mediated by NMDA, AMPA/KA channels in acute hippocampal slices: Neuroprotective effect of resveratrol	Quincozes-Santos et al.	This article is not related to a model of Parkinson’s disease development
**54**	The Use of Flavonoids in Central Nervous System Disorders	Grosso et al.	The article investigate applications of phenolic compound in neurodegenerative disorders, not for Parkinson's disease specifically
**54**	Modulating Self-Assembly of Amyloidogenic Proteins as a	Liu, T. and Bitan, G.	The article investigate applications of compounds, not specifically phenolic

	Therapeutic Approach for Neurodegenerative Diseases: Strategies and Mechanisms		compounds, in neurodegenerative disorders, not for Parkinson's disease specifically
**54**	Oxidatively generated DNA damage after Cu(II) catalysis of dopamine and related catecholamine neurotransmitters and neurotoxins: Role of reactive oxygen species	Spencer et al.	The article doesn't investigate phenolic compounds
**54**	Approaches to Prevent Dopamine Quinone-Induced Neurotoxicity	Miyazaki, I. and Asanuma, M.	The article doesn't investigate phenolic compounds
**54**	Oxidation of dopamine to aminochrome as a mechanism for neurodegeneration of dopaminergic systems in Parkinson's disease. Possible neuroprotective role of DT-diaphorase	Graumann et al.	The article doesn't investigate phenolic compounds
**54**	On the mechanism of homocysteine pathophysiology and pathogenesis: a unifying hypothesis	Zhu, B. T.	The article investigate applications of compounds, not specifically phenolic compounds, in neurodegenerative disorders, not for Parkinson's disease specifically
**53**	Emerging therapeutic potentials of dual-acting MAO and AChE inhibitors in Alzheimer's and Parkinson's diseases	Mathew et al.	The article investigate applications of phenolic compounds in Alzheimer's and Parkinson's diseases, not for Parkinson's disease specifically

**53**	Naringenin Suppresses Neuroinflammatory Responses Through Inducing Suppressor of Cytokine Signaling 3 Expression	Wu et al.	The article doesn't investigate Parkinson's disease specifically
**53**	Natural Compounds May Open New Routes to Treatment of Amyloid Diseases	Bieschke, J.	The article investigate applications of phenolic compound in amyloid diseases, not for Parkinson's disease specifically
**53**	Polyphenol-induced dissociation of various amyloid fibrils results in a methionine-independent formation of ROS	Shoval et al.	The article investigate applications of phenolic compound in dissociation of amyloid fibrils, not for Parkinson's disease specifically
**53**	Increase of antioxidative potential by tert-butylhydroquinone protects against cell death associated with 6-hydroxydopamine-induced oxidative stress in neuroblastoma SH-SY5Y cells	Hara et al.	The article doesn't investigate phenolic compounds
**53**	Recombinant cytochrome P450 2D18 metabolism of dopamine and arachidonic acid	Thompson, C. M.; Capdevila, J. H.; Strobel, H. W.	The article doesn't investigate phenolic compounds. Study different neurological disorders, not Parkinson's disease specifically
**52**	Putative Role of Red Wine Polyphenols against Brain Pathology in Alzheimer's and Parkinson's Disease	Caruana, M.; Cauchi, R. and Vassallo, N.	The article investigate applications of phenolic compounds in Alzheimer's and Parkinson's diseases, not for Parkinson's disease specifically
**52**	Recent developments on the structure-activity relationship studies of MAO inhibitors and	Kumar et al.	The article doesn't investigate phenolic compounds. Study different neurological disorders, not Parkinson's disease specifically

	their role in different neurological disorders		
**52**	The Novel Cholinesterase-Monoamine Oxidase Inhibitor and Antioxidant, Ladostigil, Confers Neuroprotection in Neuroblastoma Cells and Aged Rats	Bar-Am et al.	The article investigate applications of synthetic compounds, not specifically phenolic compounds
**52**	Toxic effects of dopamine metabolism in Parkinson's disease	Hattori et al.	The article doesn’t investigate phenolic compounds
**52**	Microwave-induced stimulation of L-DOPA, phenolics and antioxidant activity in fava bean (Vicia faba) for Parkinson's diet	Randhir, R. and Shetty, K.	The article investigate applications of compounds, not specifically phenolic compounds
**52**	Brain mitochondria catalyze the oxidation of 7-(2-aminoethyl)-3,4-dihydro-5-hydroxy-2H-1,4-benzothiazine-3-carboxylic acid (DHBT-1) to intermediates that irreversibly inhibit complex I and scavenge glutathione, potential relevance to the pathogenesis of Parkinson's disease	Li, H.; Shen, X. M. and Dryhurst, G.	The article doesn’t investigate phenolic compounds
**51**	Hesperidin as a Neuroprotective Agent: A	Hajialyan et al.	The article investigate applications of phenolic compound in neuroprotection, not for Parkinson's disease specifically

	Review of Animal and Clinical Evidence		
**51**	Inhibition of amyloid fibrillation of lysozyme by phenolic compounds involves quinoprotein formation	Feng, S.; Song, X. H. and Zeng, C. M.	This article is not related to a model of Parkinson’s disease development
**51**	Deprenyl prevents MPP+-induced oxidative damage in PC12 cells by the upregulation of Nrf2-mediated NQO1 expression through the activation of PI3K/Akt and Erk	Xiao et al.	The article doesn’t investigate phenolic compounds
**51**	Epigallocatechin gallate (EGCG) suppresses beta-amyloid-induced neurotoxicity through inhibiting c-Abl/FE65 nuclear translocation and GSK3 beta activation	Lin et al.	This article is not related to a model of Parkinson’s disease development
**51**	Tear film tests in Parkinson's disease patients	Tamer et al.	The article doesn’t investigate phenolic compounds
**51**	Ibuprofen and apigenin induce apoptosis and cell cycle arrest in activated microglia	Elsisi et al.	The article investigate applications of compounds, not specifically phenolic compounds
**51**	Adenosine A(2A) receptor enhances GABA(A)-mediated IPSCs in the rat globus pallidus	Shindou et al.	The article doesn’t investigate phenolic compounds and Parkinson’s disease
**51**	Influence of glutathione on the oxidation chemistry of the catecholaminergic neurotransmitter dopamine	Zhang, F. and; Dryhurst, G.	The article doesn’t investigate phenolic compounds

**50**	Mito-Apocynin Prevents Mitochondrial Dysfunction, Microglial Activation, Oxidative Damage, and Progressive Neurodegeneration in MitoPark Transgenic Mice	Langley et al.	The article doesn't investigate phenolic compounds
**50**	L-dopa-induced dopamine synthesis and oxidative stress in serotonergic cells	Stansley, B. J. and Yamamoto, B. K.	The article doesn't investigate phenolic compounds
**50**	NMR Characterization of Monomeric and Oligomeric Conformations of Human Calcitonin and Its Interaction with EGCG	Huang et al.	The article doesn't investigate Parkinson's disease
**49**	Neuroprotective Effect of Quercetin Against the Detrimental Effects of LPS in the Adult Mouse Brain	Khan et al.	This article is not related to a model of Parkinson's disease development
**49**	Inhibition of protein misfolding and aggregation by natural phenolic compounds	Dhouafli et al.	The article investigate applications of phenolic compounds in inhibition of protein misfolding and aggregation, not for Parkinson's disease specifically
**49**	Neuroprotection through flavonoid: Enhancement of the glyoxalase pathway	Frandsen, J. R. and Narayanasamy, P.	The article investigate applications of phenolic compounds in neuroprotection, not for Parkinson's disease specifically
**49**	Privileged scaffolds as MAO inhibitors: Retrospect and prospects	Tripathi et al.	The article investigate applications of compounds, not specifically phenolic compounds, in MAO inhibition, not for Parkinson's disease specifically

**49**	Apigenin as neuroprotective agent: Of mice and men	Nabavi et al.	The article investigate applications of phenolic compound in neuroprotection, not for Parkinson's disease specifically
**49**	Activation of endogenous antioxidants as a common therapeutic strategy against cancer, neurodegeneration and cardiovascular diseases: A lesson learnt from DJ-1	Chan, J. Y. H. and Chan, S. H. H.	The article investigate applications of antioxidant compounds, not specifically phenolic compounds, in several diseases, not for Parkinson's disease specifically
**49**	Neuroprotective Effects of Rutin in Streptozotocin-Induced Diabetic Rat Retina	Ola et al.	This article is not related to a model of Parkinson’s disease development
**49**	Anti-inflammatory Activity of a Honey Flavonoid Extract on Lipopolysaccharide-Activated N13 Microglial Cells	Candiracci et al.	The article investigate applications of phenolic compounds in neuroprotection, not for Parkinson's disease specifically
**49**	Genetic modification of the association of paraquat and Parkinson's disease	Goldman et al.	The article doesn’t investigate phenolic compounds
**49**	Neuroprotective and neurotoxic roles of levodopa (L-DOPA) in neurodegenerative disorders relating to Parkinson's disease	Kostrzewa, R. M.; Kostrzewa, J. P. and Brus, R.	The article doesn’t investigate phenolic compounds
**48**	Dopamine, Oxidative Stress and Protein-Quinone Modifications in Parkinson's and Other Neurodegenerative Diseases	Monzani et al.	The article doesn’t investigate phenolic compounds

**48**	Dietary PolyphenolsImportant Non-Nutrients in the Prevention of Chronic Noncommunicable Diseases. A Systematic Review	Koch, W.	The article doesn't investigate phenolic compounds and study neurodegenerative diseases, not Parkinson's disease specifically
**48**	Bamboo: A rich source of natural antioxidants and its applications in the food and pharmaceutical industry	Nirmala et al.	The article investigate applications of antioxidant compounds, not specifically phenolic compounds, in pharmaceutical industry, not for Parkinson's disease specifically
**48**	Neuroprotective Effects of Citrus Fruit-Derived Flavonoids, Nobiletin and Tangeretin in Alzheimer's and Parkinson's Disease	Braidy et al.	The article investigate applications of phenolic compounds in Alzheimer's and Parkinson's Disease, not for Parkinson's disease specifically
**48**	Chocolate consumption and risk of stroke A prospective cohort of men and meta-analysis	Larsson, S. C.; Virtamo, J. and Wolk, A.	This article is not related to a model of Parkinson's disease development
**48**	Olive oil reduces oxidative damage in a 3-nitropropionic acid-induced Huntington's disease-like rat model	Tasset et al.	This article is not related to a model of Parkinson's disease development
**48**	Alpha-tocopherol quinone inhibits beta-amyloid aggregation and cytotoxicity, disaggregates preformed fibrils and decreases the production of reactive oxygen species, NO and inflammatory cytokines	Yang et al.	This article is not related to a model of Parkinson's disease development

**48**	Pramipexole protects against H2O2-induced PC12 cell death	Fujita et al.	The article doesn't investigate phenolic compounds
**48**	Cervical dystonia - Pathophysiology and treatment options	Velickovic, M.; Benabou, R. and Brin, M. F.	The article doesn't investigate phenolic compounds and study cervical dystonia diseases, not Parkinson's disease specifically
**48**	Antibodies from patients with Parkinson's disease react with protein modified by dopamine oxidation	Rowe et al.	The article doesn't investigate phenolic compounds
**48**	Secondary excitotoxicity contributes to dopamine-induced apoptosis of dopaminergic neuronal cultures	Zhang et al.	The article doesn't investigate phenolic compounds
**47**	The Effects of Flavonoids on Cardiovascular Health: A Review of Human Intervention Trials and Implications for Cerebrovascular Function	Rees, A.; Dodd, G. F. and Spencer, J. P. E.	The article investigate applications of phenolic compounds in cerebrovascular function, not for Parkinson's disease specifically
**47**	A review on flavonoid-based scaffolds as multi-target-directed ligands (MTDLs) for Alzheimer's disease	Jalili-Baleh et al.	This article is not related to a model of Parkinson's disease development
**47**	A review on traditional uses, phytochemistry, pharmacology, pharmacokinetics and toxicology of the genus Peganum	Li, S.; Cheng, X. and Wang, C.	The article doesn't investigate phenolic compounds and study several aspects of genus Peganum, not Parkinson's disease specifically

**47**	Cognitive and neuroprotective effects of chlorogenic acid	Heitman, E. and Ingram, D. K.	The article investigate applications of phenolic compound cognitive and neuroprotective effects, not for Parkinson's disease specifically
